# A systematic analysis of gene–gene interaction in multiple sclerosis

**DOI:** 10.1186/s12920-022-01247-3

**Published:** 2022-04-30

**Authors:** Lotfi Slim, Clément Chatelain, Hélène de Foucauld, Chloé-Agathe Azencott

**Affiliations:** 1 CBIO, MINES ParisTech, PSL Research University, 75006 Paris, France; 2 Translational Sciences, SANOFI R&D, 91385 Chilly-Mazarin, France; 3 NVIDIA Corporation, Santa Clara, 95051 USA; 4 Institut Curie, PSL Research University, 75005 Paris, France; 5 U900, Inserm, 75005 Paris, France

**Keywords:** GWAS, Epistasis, Multiple sclerosis, Gene–gene interaction, Causal inference

## Abstract

**Background:**

For the most part, genome-wide association studies (GWAS) have only partially explained the heritability of complex diseases. One of their limitations is to assume independent contributions of individual variants to the phenotype. Many tools have therefore been developed to investigate the interactions between distant loci, or epistasis. Among them, the recently proposed EpiGWAS models the interactions between a target variant and the rest of the genome. However, applying this approach to studying interactions along all genes of a disease map is not straightforward. Here, we propose a pipeline to that effect, which we illustrate by investigating a multiple sclerosis GWAS dataset from the Wellcome Trust Case Control Consortium 2 through 19 disease maps from the MetaCore pathway database.

**Results:**

For each disease map, we build an epistatic network by connecting the genes that are deemed to interact. These networks tend to be connected, complementary to the disease maps and contain hubs. In addition, we report 4 epistatic gene pairs involving missense variants, and 25 gene pairs with a deleterious epistatic effect mediated by eQTLs. Among these, we highlight the interaction of GLI-1 and SUFU, and of IP10 and NF-$\kappa$B, as they both match known biological interactions. The latter pair is particularly promising for therapeutic development, as both genes have known inhibitors.

**Conclusions:**

Our study showcases the ability of EpiGWAS to uncover biologically interpretable epistatic interactions that are potentially actionable for the development of combination therapy.

**Supplementary Information:**

The online version contains supplementary material available at 10.1186/s12920-022-01247-3.

## Background

The development of Genome-Wide Association Studies (or GWAS) has made it possible to explore the genetic causes of the heritability of complex diseases. In such studies, large cohorts of cases and controls are jointly studied in order to discover loci associated with the disease. This is typically achieved through a series of univariate statistical tests of association between a Single Nucleotide Polymorphism (SNP) and the phenotype [1]. Though the statistical validity of this approach is indisputable, it suffers from a lack of statistical power because of high dimensionality and multiple hypothesis testing [2]. Another limitation of this approach is that the lack of direct biological explanations for the significant SNPs hinders the interpretability of GWAS.

In addition, single-locus analyses, by design, do not take into account interactions between distinct genes, or epistasis [3]. This is restrictive because genes do not act in isolation but interact with each others. In recent years, many approaches to epistasis detection have been proposed. Among them, the recently proposed EpiGWAS [4] focuses on interactions between a specific SNP and the rest of the genome. This contrasts with the more frequent strategy of exhaustive pairwise testing. The target SNP is chosen on the basis of an already established linked with the phenotype, which facilitates interpretation and reduces the number of interactions to study. In addition, rather than studying interactions between two SNPs, EpiGWAS models the interaction of the target SNP with all other variants in the genome at once.

In practice, one is likely to be interested in querying more than one target SNP. A possible source of target SNPs is so-called disease maps, that is to say high-quality, expert-curated representations of the mechanisms involved in a disease. Disease maps contain signalling, metabolic and gene regulatory pathways and can be represented as pairs of interacting genes. Any SNP connected to these genes is therefore a reasonable target SNP. In this article, we show how to apply EpiGWAS with a set of disease maps to identify pairs of genes that are likely to be interacting towards the disease of interest. We illustrate our approach on a case study of multiple sclerosis (MS), a chronic disease damaging the central nervous system [5].

A number of marketed drugs [6] attenuate the symptoms of multiple sclerosis. However, an efficient drug targeting its root causes is still elusive. This is partially due to our limited understanding of the mechanisms governing the diseases. Several studies have demonstrated that heritability is a major component in multiple sclerosis [7, 8], motivating the use of GWAS to study it. At least fourteen GWAS on multiple sclerosis have been published so far [9], identifiying hundreds of loci [10, 11] statistically associated with the disease. The biology behind some of these loci has been clarified [12–14], although it remains unexplained for the majority of associated loci [9].

At least two gene–gene interactions have been discovered in multiple sclerosis: high levels of c-Jun may cause enhanced myelinating potential in Fbxw7 [15], and DDX39B is both a potent activator of IL7R exon6 splicing and a repressor of sIL7R [16]. An additional tripartite genic interaction has also been reported [17]: epistasis between the HLA-DRB1, HLA-DQA1, and HLA-DQB1 loci increases susceptibility. This further motivates the study of epistasis to understand the genetic basis of multiple sclerosis.

In this article, we use EpiGWAS on the multiple sclerosis GWAS from the Wellcome Trust Case Control Consortium 2 [18] to score interactions between all pairs of genes contained in 19 multiple sclerosis disease maps from MetaCore [19]. Our analysis yields 4 gene pairs involving missense variants, and 25 gene pairs with epistasis mediated by eQTLs. Among these interactions, two are already known: the direct binding interaction between GLI-I and SUFU, involved in oligodendrocyte precursor cells differentiation, and the regulation of IP10 transcription by NF-$\kappa$B. This confirms the capacity of the statistical study of epistasis to detect biological interactions that further our understanding of disease mechanisms.

## Methods

### Data

#### GWAS data from WTCCC2

The Wellcome Trust Consortium Case Control 2 (WTCCC2) multiple sclerosis data set consists of 9 772 cases and 17 376 controls analyzed with the Illumina Human 660-Quad and Illumina 1.2M platforms. All data sets are composed of samples of European descent, but hailing from 15 different countries. The presence of population structure, confirmed by a genomic inflation factor (GIF) of 3.72, is poised to lead to inference issues. To avoid this problem, we restrict ourselves to Caucasian British samples in both cases and controls. The resulting dataset consists of 2 048 cases and 5 733 controls with a GIF of 1.06, which proves the homogeneity of the dataset. The selected controls come from two distinct cohorts from the UK Blood Services (NBS) and the 1958 British Birth Cohort (58C). The imbalance between the numbers of cases and controls may distort the results. We therefore randomly subsample controls to obtain a number of controls equal to the number of cases. We also note that we discarded the samples singled out for quality control by the WTCCC, as well as the low quality SNPs as flagged by the WTCCC.

#### Disease maps from MetaCore

MetaCore is a commercial resource containing high-quality, manually curated biological pathway data from peer-reviewed literature. This information is organized in pathway maps, which are made of potentially multi-step interactions defining a well-established signaling mechanism. Each step is experientially validated and accepted in the research field.

Pathology maps, or disease maps, are maps created specifically for a disease mechanism. We present in Table 1 the full list of the 19 multiple sclerosis disease maps we used. The number of genes within each map greatly varies. It ranges from 13 genes for disease map (DM3305) to 100 genes (DM4593).

**Table 1:** Titles and internal IDs of MetaCore disease maps related to MS

Internal ID	Title
3302	Notch signaling in oligodendrocyte precursor cell differentiation in multiple sclerosis
3305	SHH signaling in oligodendrocyte precursor cells differentiation in multiple sclerosis
3306	Inhibition of oligodendrocyte precursor cells differentiation by Wnt signaling in multiple sclerosis
4455	Inhibition of remyelination in multiple sclerosis: regulation of cytoskeleton proteins
4593	Axonal degeneration in multiple sclerosis
4693	Role of Thyroid hormone in regulation of oligodendrocyte differentiation in multiple sclerosis
4703	Demyelination in multiple sclerosis
4791	Role of CNTF and LIF in regulation of oligodendrocyte development in multiple sclerosis
4794	Retinoic acid regulation of oligodendrocyte differentiation in multiple sclerosis
4843	Growth factors in regulation of oligodendrocyte precursor cells proliferation in multiple sclerosis
4846	Growth factors in regulation of oligodendrocyte precursor cells survival in multiple sclerosis
4901	Inhibition of remyelination in multiple sclerosis: role of cell-cell and ECM-cell interactions
5199	Cooperative action of IFN-$\gamma$ and TNF-$\alpha$ on astrocytes in multiple sclerosis
5288	Impaired inhibition of Th17 cell differentiation by IFN-$\beta$ in multiple sclerosis
5378	Role of IFN-$\beta$ in the improvement of blood-brain barrier integrity in multiple sclerosis
5398	Role of IFN-$\beta$ in activation of T cell apoptosis in multiple sclerosis
5518	Role of IFN-$\beta$ in inhibition of Th1 cell differentiation in multiple sclerosis
5601	IL-2 as a growth factor for T cells in multiple sclerosis
5611	Role of IL-2 in the enhancement of NK cell cytotoxicity in multiple sclerosis

**Fig. 1:**
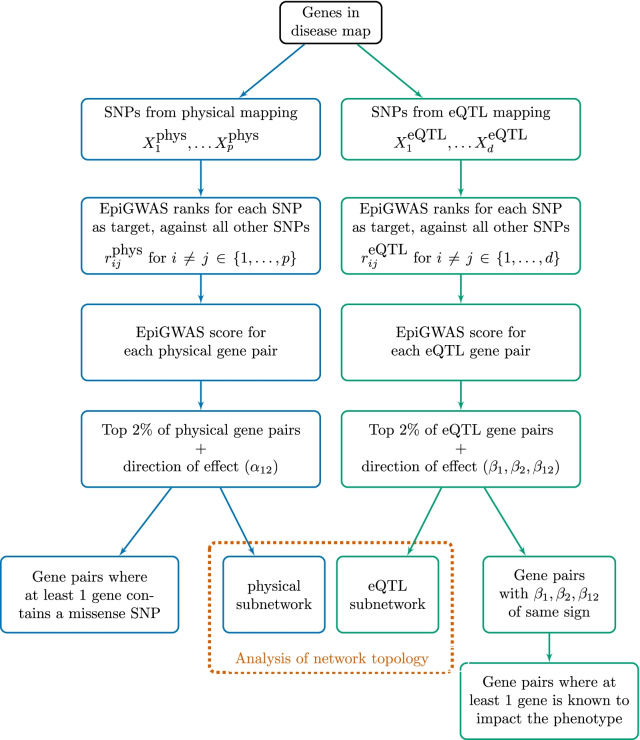
Epistatic interaction discovery pipeline

We now detail our processing pipeline. An overview is given in Fig. 1.

### Variant filtering

The first step of our study is to map SNPs to the genes involved in the 19 multiple sclerosis maps. For this purpose, we considered two kinds of mapping, so as to facilitate functional interpretation:

- *Physical mapping,* which corresponds to retrieving all genotyped SNPs located on a given gene. We use the accompanying R package metabaser [20] to first define the boundaries of a given gene, and then subset all SNPs according to their positions, as referenced in dbSNP version 144 [21].
- *eQTL mapping,* which corresponds at mapping a SNP to all genes that it is known to regulate (in *cis*). For this purpose, we used the *cis*-eQTL dataset from the eQTLGen consortium [22], which provides for each gene a list of significant eQTL-SNPs. The dataset combines 31 684 whole blood samples from 37 cohorts.

For the present study, we limit ourselves to *cis*-eQTLs and ignore *trans*-eQTLs because of their higher degree of association to gene expression. The higher association can be attributed to the proximity of the SNPs to the genes: *cis*-eQTL are located within 1 Mb window from a gene and they often closely map to either the transcription start site or the transcription end site of a gene.

Even though the two analyses are unrelated and use different sets of SNPs, some concordance for the top-scoring genes is to be expected. In fact, for the eQTLGen consortium, Võsa et al. [22] show that out of $15\,317$ trait-associated SNPs, $15.2\%$ were in high LD with the lead eQTL SNP showing the strongest association for a cis-eQTL gene. Although the mentioned association is far from perfect, it demonstrates the often-overlooked link between the two analyses.

### SNP-level epistasis detection with EpiGWAS

EpiGWAS [4] is a framework for targeted epistasis that scores interactions between a given SNP, denoted by *A*, and a set of *p* SNPs $X = \lbrace X_1,\ldots , X_p\rbrace$. *X* can cover either the whole genome or a predetermined region. Using a binary encoding for *A*: $A\in \lbrace -1, +1\rbrace$, the phenotype *Y* is written as

1 $$\begin{aligned} Y = \mu (X) + \delta (X) \cdot A + \epsilon , \end{aligned}$$

where $\epsilon$ is a zero-mean random variable and

2 $$\begin{aligned} \left\{ \begin{aligned} \mu (X)&= \frac{1}{2}\left( {\mathbb {E}}(Y|A=+1,X)+{\mathbb {E}}(Y|A=-1,X)\right) \,\\ \delta (X)&= \frac{1}{2}\left( {\mathbb {E}}(Y|A=+1,X)-{\mathbb {E}}(Y|A=-1,X)\right) \,. \end{aligned} \right. \end{aligned}$$

The first term in Eq. 2, $\mu (X)$, models the average effect of the target *A* on the expected phenotype, conditionally on *X*. The second term, $\delta (X)$, accounts for the interactions between *A* and *X*. If $\delta (X)$ is sparse, that is to say, that it only depends on a subset of the *p* elements of *X*, then the SNPs in this subset are the ones interacting with *A*.

The estimation of $\delta (X)$ is made difficult by the fact that for any given sample, only one of the two cases $A=+1$ and $A=-1$ is observed. EpiGWAS leverages on the literature of causal inference on observational data [23] to conduct this estimation. In observational studies and particularly in clinical trials, only one of the two states of the treatment *A* is observed and a different encoding has to be applied to estimate the term $\delta (X)$. Indeed, using for the target SNP an encoding in $\lbrace 0, 1\rbrace$, obtained as ${\tilde{A}} = (A + 1)/2,$ we can rewrite $\delta (X)$ as

$$\begin{aligned} \delta (X) = \frac{1}{2}{\mathbb {E}}\left[ Y\left( \frac{{\tilde{A}}}{{\mathbb {P}}({\tilde{A}}=1|X)}-\frac{1-{\tilde{A}}}{{\mathbb {P}}({\tilde{A}}=0|X)}\right) {\Bigg |} X\right] \,. \end{aligned}$$

This suggests obtaining a sparse model for $\delta (X)$ by using a penalized regression approach (in EpiGWAS, an elastic net) to fit a sparse linear model between the *modified outcome*

$$\begin{aligned} {\tilde{Y}} = Y\left( \frac{{\tilde{A}}}{{\mathbb {P}}({\tilde{A}}=1|X)} - \frac{1-{\tilde{A}}}{{\mathbb {P}}({\tilde{A}}=0|X)}\right) \end{aligned}$$

and *X*. The regression coefficient of SNP $X_i$ in this sparse linear model can then be interpreted as a score of the interaction between this SNP and target SNP *A*,  that accounts for interactions between *A* and all SNPs in *X*.

To compute the modified outcome, we need an estimate of the propensity scores ${\mathbb {P}}({\tilde{A}}=1|X)$. This can be achieved using the hidden Markov Model from fastPHASE [24]. In this model, the observed states correspond to SNPs and the hidden states correspond to structural dependence states. After fitting this model in a chromosome by chromosome fashion, the propensity scores are obtained through application of the forward algorithm [25].

If the estimation error of ${\mathbb {P}}(A|X)$ is large or severe overfitting occurs, the use of the inverse of the estimated scores can result in numerical instability and bias the results. EpiGWAS addresses this issue by resorting to an adjustment of the modified outcome called the robust modified outcome. This approach is known to have small large-sample variance in the causal inference literature, and was empirically superior to other corrections [4].

### From SNP-level EpiGWAS scores to gene-level epistasis scores

Given a set of *p* SNPs $X_1, X_2, \ldots , X_p$ mapped to the genes of a given disease map, we use EpiGWAS to obtain scores for the interaction of each of the SNPs in $\{X_1, \ldots , X_{i-1}, X_{i+1}, \ldots , X_p\}$ with target SNP $X_i.$ The interpretability and usability of such an output is limited, because of the large number of interactions, but also because scores obtained against different target SNPs are not comparable, as the set of SNPs in *X* is different for each target *A*. Furthermore, despite their robustness, these scores have limited biological meaning.

A first step to improve interpretability is to use rankings. From a practical point of view, rankings are a sensible choice because only the highest-ranking SNPs are used. Rankings also improve comparability between different targets because of the similarity of scale and insensitivity to the underlying parameterization. For a target SNP *i*, we therefore denote by $r_{ij}\in \lbrace 1, \ldots , p-1\rbrace$ the rank of the score of SNP *j* among all scores against target SNP *i*, in decreasing order.

Another immediate benefit of the use of rankings is the possibility of combining different rankings. For example, for two SNPs *i* and *j*, we can define the following epistasis interaction score:

3 $$\begin{aligned} {{\,\mathrm{inter}\,}}(i, j) = \frac{1}{\sqrt{r_{ij} + r_{ji}}}. \end{aligned}$$

The interaction score in Eq. 3 has the advantages of symmetry and boundedness. The scores are comprised between $\frac{1}{\sqrt{2(p-1})}$ and $\frac{1}{2}$. If $p=1\,000,$ this corresponds to a range of $0.02 - 0.05$. Additionally, combining two pairwise scores $r_{ij}$ and $r_{ji}$ can help control the estimation errors for one of the targets. For example, if two SNPs *i* and *j* are in interaction and the result $r_{ij}$ is not sufficiently high to reflect that, a good ranking of $r_{ji}$ can help compensate this effect.

We can further aggregate the rankings to detect interactions between genes. More generally, the rankings can be combined to detect interactions between any disjoint sets of SNPs, such as biological pathways or regulatory regions. Let $p'$ be the total number of genes in the disease map, and let us denote by $G_{i^\prime }$ the set of SNPs mapped to gene $i^\prime$. The interaction score between two genes $i^\prime$ and $j^\prime$ can be computed by considering all pairwise scores between SNPs mapped to $i^\prime$ and SNPs mapped to $j^\prime$, and we define

4 $$\begin{aligned} {{\,\mathrm{inter}\,}}(G_{i^\prime }, G_{j^\prime }) = \frac{1}{|G_{i^\prime }||G_{j^\prime }|} \sum \limits _{i\in G_{i^\prime }} \sum \limits _{j\in G_{j^\prime }} \frac{1}{\sqrt{r_{ij} + r_{ji}}}. \end{aligned}$$

Thanks to the symmetry of SNP-SNP scores in Eq. 3, the gene–gene scores in Eq. 4 are symmetric, too. Averaging over all SNPs mapped to the genes reduces the impact of the size of the genes.

### Direction of the epistatic effect

An epistatic interaction can be either positive or negative, depending on whether it increases or decreases disease susceptibility. To study the direction of this effect, we focus on the top-scoring pair of SNPs, as it has the largest effect on the global gene–gene score. More specifically, following [26], for a binary outcome *Y* and two explanatory variables $X_1$ and $X_2$, the direction of the epistatic effect is given by the sign of the interaction coefficient $\alpha _{12}$ in the logistic model

5 $$\begin{aligned} {{\,\mathrm{logit}\,}}{\mathbb {P}}(Y|X_1, X_2) = \alpha _0 + \alpha _1 X_1 + \alpha _2 X_2 + \alpha _{12} X_1 X_2. \end{aligned}$$

When the SNPs have been obtained through eQTL mapping, this methodology can be refined. Indeed, the effect of SNP $X_i$ on the expression level $e_{i}$ of the gene $G_{i}$ to which it is mapped can be modeled as $e_{i} = \gamma _{i} + \beta _{i} X_i$. The direction of the epistatic effect between genes $G_1$ and $G_2$ can be deduced from the sign of the following ratio:

6 $$\begin{aligned} {{\,\mathrm{dir}\,}}(G_1, G_2) = {{\,\mathrm{sign}\,}}\frac{\alpha _{12}}{\beta _1\cdot \beta _2}. \end{aligned}$$

Indeed, the logistic model in Eq (5) becomes

7 $$\begin{aligned} {{\,\mathrm{logit}\,}}{\mathbb {P}}(Y|X_1, X_2)= & {} \,\alpha _0 + \alpha _1 \frac{e_1 - \gamma _1}{\beta _1} + \alpha _2 \frac{e_2 - \gamma _2}{\beta _2}\nonumber \\&+ \frac{\alpha _{12}}{\beta _1\cdot \beta _2} (e_1 - \gamma _1) (e_2 - \gamma _2) \end{aligned}$$

and the ratio of Eq. (6) indeed governs the effect on the phenotype of the interaction between the expression of the two genes.

To the best of our knowledge, this is the first study which studies epistasis from such a perspective by including eQTL scores in this way and by moving back and forth between SNP-level and gene-level epistasis. Furthermore, the synergy score in Eq. 6 can also be interpreted as an extension of Mendelian randomization [27] to second-order interaction effects.

The eQTLGen consortium [22] does not directly supply the effect sizes $\beta _1$ and $\beta _2$ in the linear expression models. For each SNP, the effect size $\beta$ is derived from the corresponding Z-score using the following relationship:

8 $$\begin{aligned} \beta = \frac{Z}{\sqrt{2\,q(1-q)\,(m+Z^2)}}, \end{aligned}$$

where *q* is the MAF of the SNP of interest, as reported in the 1kG v1p3 ALL reference panel and *m* is the cohort size.

### Significance of network topological properties

A list of epistatic interactions among a set of genes can be represented as network where the vertices are the genes and each gene corresponds to an epistatic interaction. We can then observe some topological properties of these networks, such as whether they form a single connected component; whether they contain nodes of high degree; or whether they correspond to known biological interactions.

To evaluate whether these properties are significant or are likely to happen by chance, we propose a permutation procedure. More particularly, to evaluate the significance of an epistatic network of *m* edges (over a total of *n* genes considered) forming a single connected component, we sample *S* times *m* pairs of vertices from a set of *n* vertices. Denoting by $S^\prime$ the number of times these *m* pairs also form a connected component, we then compute the p-value as $S^\prime /S.$

To evaluate whether the epistatic network contains nodes of high degree, we compute its maximum node degree $d_{\max }$, and repeat the above procedure, denoting by $S^\prime$ the number of times the *m* sampled pairs form a network with maximal degree at least as large as $d_{\max }$.

## Results

We exhaustively compute the gene–gene interaction scores of Eq. 4 to obtain $p^\prime (p^\prime -1)/2$ interaction scores per disease map, where $p^\prime$ is the number of genes in the map. Given the size of the maps (see Table 1), the interpretation of the full results is rather difficult. We instead focus on the $2\%$ top-scoring pairs for the two analyses. Our rationale is that this is a number large enough to retain at least one gene pair on each map, without yielding so many pairs that their study is made difficult.

**Table 2:** Number of SNPs, genes, gene pairs and top 2% of gene pairs for each mapping and disease map

Map ID	Physical mapping				eQTL mapping			
	#SNPs	#genes	#gene pairs	top 2%	#SNPs	#genes	#gene pairs	top 2%
3302	416	21	210	4	833	19	171	3
3305	70	10	45	1	238	8	28	1
3306	383	21	210	4	869	19	171	3
4455	755	38	703	14	1813	36	630	13
4593	1295	24	276	6	1647	17	136	3
4693	544	34	561	11	912	27	351	7
4703	331	28	378	8	999	27	351	7
4791	252	24	276	6	1264	23	253	5
4794	84	15	105	2	331	12	66	1
4843	984	32	496	10	1401	29	406	8
4846	1318	36	630	13	1555	32	496	10
4901	1173	35	595	12	1209	24	276	6
5199	656	28	378	8	1320	32	496	10
5288	515	27	351	7	724	22	231	5
5378	257	22	231	5	907	22	231	5
5398	141	21	210	4	1050	24	276	6
5518	392	29	406	8	1474	27	351	7
5601	348	28	378	8	742	25	300	6
5611	224	22	231	5	906	24	276	6

It would of course be preferable here to use p-values for the significance of the interactions, but there does not seem to be any other way than permutation testing to obtain those, and this is computationally unfeasible. Indeed, when evaluating the 703 gene pairs of DM4455, one would require a significance level of, for example, $\alpha = 0.05/703$ (accounting for multiple hypotheses). The number of permutations would have to be greater than $1/\alpha$, so around at least 15 000 in our example, which is out of reach.

We show in Additional file 1: Appendix A that the results obtained do not vary much with the choice of strategy to select a small number of pairs per map.

### eQTL mapping yields more SNP pairs to test than the physical mapping

The number of SNPs obtained through eQTL mapping is larger than that obtained through physical mapping: the median number of SNPs per disease map is 392 for the physical mapping analysis and 999 for the eQTL-mapping analysis. In Table 2, we give the exact number of SNPs per disease map for each type of mapping.

**Table 3:** Connectivity: whether the epistatic interactions form a single connected component, for the networks obtained by physical mapping, eQTL mapping, and joining both

Map ID	Physical mapping (*p*-value)	eQTL mapping (*p*-value)	Joint (*p*-value)
3302	Yes (**0.035**)	Yes (0.077)	Yes (**0.020**)
3305	Yes (1.000)	Yes (1.000)	Yes (0.377)
3306	Yes (**0.034**)	Yes (0.078)	Yes (**0.016**)
4455	Yes (**0.000**)	Yes (**0.001**)	Yes (**0.018**)
4593	Yes (**0.009**)	Yes (0.092)	No (NA)
4693	Yes (**0.001**)	Yes (**0.006**)	Yes (**0.006**)
4703	Yes (**0.004**)	Yes (**0.005**)	Yes (**0.011**)
4791	Yes (**0.013**)	Yes (**0.018**)	Yes (**0.014**)
4794	Yes (0.256)	Yes (1.000)	Yes (0.098)
4843	No (NA)	Yes (**0.004**)	Yes (**0.009**)
4846	Yes (**0.001**)	Yes (**0.002**)	Yes (**0.017**)
4901	Yes (**0.001**)	Yes (**0.009**)	Yes (**0.004**)
5199	Yes (**0.003**)	Yes (**0.001**)	Yes (**0.011**)
5288	Yes (**0.005**)	Yes (**0.014**)	Yes (**0.012**)
5378	Yes (**0.018**)	Yes (**0.023**)	Yes (**0.018**)
5398	Yes (**0.032**)	Yes (**0.011**)	Yes (**0.014**)
5518	Yes (**0.004**)	Yes (**0.005**)	Yes (**0.011**)
5601	Yes (**0.004**)	Yes (**0.008**)	Yes (**0.013**)
5611	Yes (**0.018**)	Yes (**0.012**)	Yes (**0.015**)

The bold values correspond to* p*-values below the significance threshold of 0.05

### Topology of gene–gene interactions detected with EpiGWAS

**Fig. 2:**
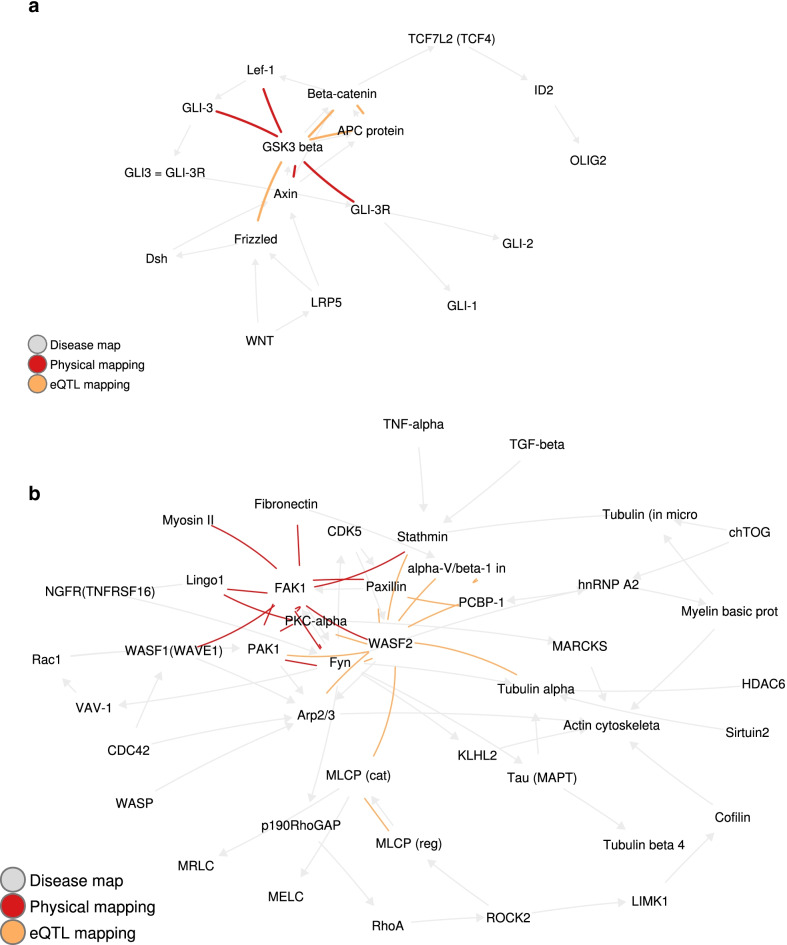
Examples of epistatic pairs detected on two disease maps. **a** The 2% top-scoring pairs in DM 3306 for eQTL and physical mappings. **b** The 2% top-scoring pairs in DM 4455 for eQTL and physical mappings

Figure 2 shows the epistatic interactions we obtain for two disease maps. All other maps can be found in Additional file 1: Appendix B. These two examples illustrate properties we observe more generally across all disease maps and that we discuss in more details below: connectedeness (the epistatic pairs form connected components), complementarity (of the epistatic interactions obtained through eQTL mapping with the interactions obtained through physical mapping, as well as the epistatic interactions with the known biological interactions), and high centrality of some nodes in those networks.

#### Gene–gene epistatic interactions form connected components

**Fig. 3:**
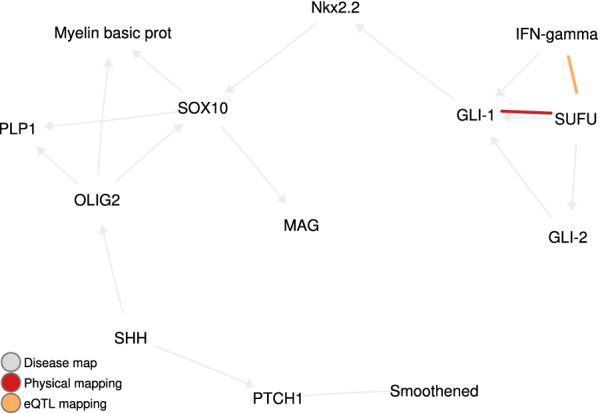
The $2\%$ top-scoring pairs in DM 3305 for eQTL and physical mappings. Note the GL1–SUFU pair

Most of the epistatic interaction networks we obtained form a single connected component, as seen for example on Fig. 2b. The only exceptions are DM 4834 for the epistatic network obtained through physical mapping (two components, one of which is formed by a single edge) and DM 4593 for the union of the epistatic networks obtained through physical and eQTL mappings (each of the networks form a separate connected component).

**Fig. 4:**
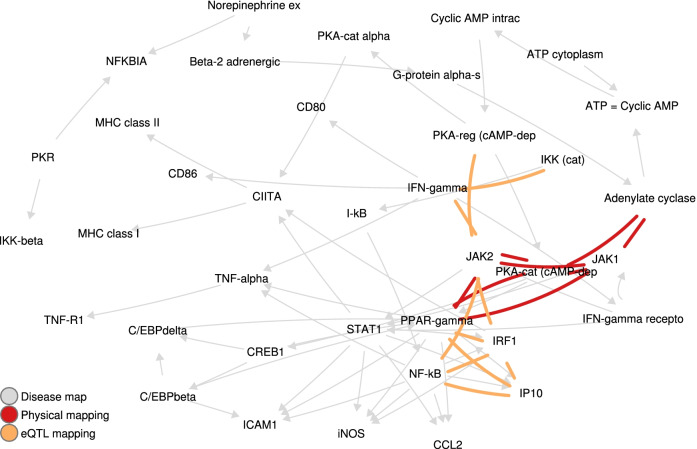
The $2\%$ top-scoring pairs in DM 5199 for eQTL and physical mappings. Note the NF-$\kappa$B – IP10 pair

We report in Table 3 the significance of these observations (see “Significance of network topological properties” section). Given the number of nodes in the maps and epistatic edges selected between them, the only cases where it can be expected to obtain a single connected component are those where the number of selected edges is lower than 3 (DM 3305 with a single edge for either mapping, DM 4593 with 3 edges for the eQTL mapping, and DM 4794 with 2 edges for the physical mapping and 1 edge for the eQTL mapping). Hence all large connected components, obtained on larger maps, are significant.

**Table 4:** Centrality: Node(s) of maximum degree in the epistatic network obtained by joining the physical epistatic network and the eQTL epistatic network

Map ID	Max node degree	*p*-value	Node(s) of max degree
3302	4	**0.033**	ADAM17, CNTN1 (F3)
3305	2	0.365	SUFU
3306	6	**0.000**	GSK3 beta
4455	11	**0.000**	WASF2
4593	4	0.086	NCX1
4693	10	**0.000**	mTORC1
4703	5	0.056	AKT(PKB), Caspase-8
4791	5	**0.018**	AKT(PKB), PI3K reg class IA
4794	2	0.553	DHA2, GALC
4843	8	**0.000**	SHP-2
4846	11	**0.000**	Neuregulin 1
4901	12	**0.000**	FAK1
5199	7	**0.001**	JAK2
5288	6	**0.001**	IL-1RI, ROR-alpha
5378	5	**0.013**	JNK(MAPK8-10)
5398	6	**0.001**	TRADD
5518	6	**0.004**	AKT(PKB)
5601	7	**0.000**	Bcl-XL
5611	5	**0.018**	Granzyme B, KLRK1 (NKG2D)

The bold values correspond to* p*-values below the significance threshold of 0.05

Such large connected components indicate joint effects in large sets of SNPs. They point to a multiplicity of ways in which the same biological process may be perturbed to result in multiple sclerosis, capturing the complex architecture of the disease. This suggests new drug development strategies against multiple sclerosis, either by targeting the most druggable gene in the component, or by targeting several of its genes through combination therapy so as to maximize effect on the biological process.

Connectedness and centrality in epistatic networks was already observed at the SNP level for other phenotypes with a different methodological approach (see for example [28]).

#### eQTL and physical mappings yield complementary gene–gene epistatic interactions

With the exception of DM 4593, the epistatic networks obtained through the two mappings are connected, that is to say, they share at least one common node/gene. In fact, they are often connected through multiple nodes, without an overlap in edges. This can again be observed for example on Fig. 2b. The last column of Table 3 shows that this connection between the two epistatic networks is not expected by chance.

**Table 5:** Pairs of genes identified by physical mapping, and selected because at least one of the SNPs involved has a direct consequence as protein dysfunction

Map ID	Gene pair	Type of interaction
3305	GLI-1 and SUFU	Direct interaction between the genes
		Uunspecified impact on MS
4703	AKT (PKB) and MEKK1 (MAP3K1)	No direct interaction between the genes
		AKT has a specified impact on MS
5611	Granzyme B and KLRK1 (NKG2D)	No direct interaction between the genes
		Unspecified impact on MS
	Granzyme B and PI3K cat class IA	No direct interaction between the genes
		Unspecified impact on MS

**Fig. 5:**
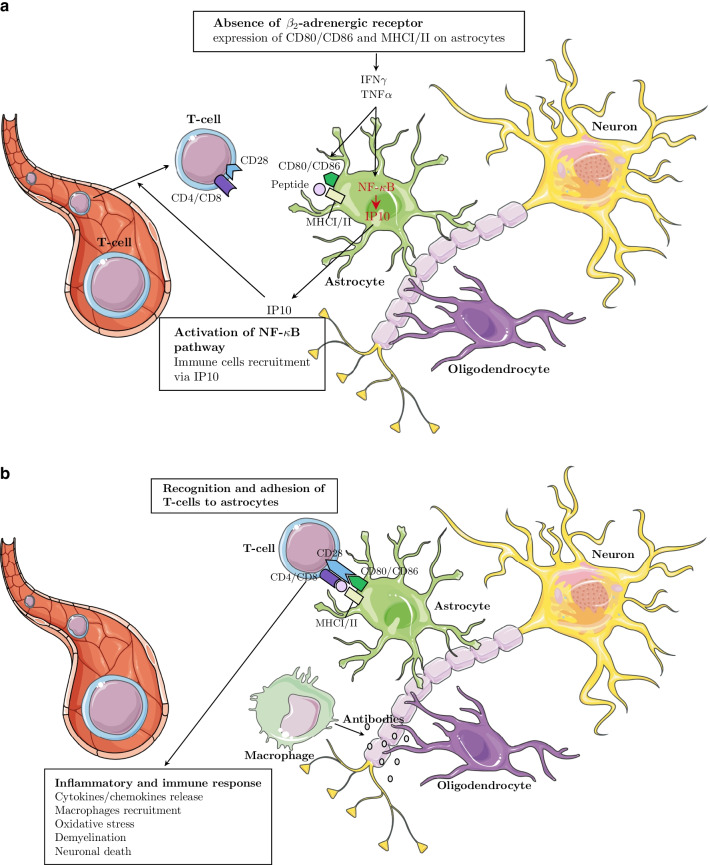
Schematic representation of the role played by the gene pairs NF-$\kappa$B/IP10 in the development of demyelination in MS. **a** Transformation of astrocytes in immuno-competent cells and T-cells recruitment following the NF-B/IP10 axis activation in MS. **b** After recruitment of T-cells, adhesion of T-cell/astrocyte leads to in ammatory and immune response inducing neuron damage

Therefore, the two types of mappings recover distinct, though connected, interactions. This suggests that they yield complementary information about the genetic architecture of the disease.

We therefore consider the union of the two epistatic networks for further study.

#### Gene–gene epistatic interaction networks contain hubs

We report in Table 4 the maximum degree in the gene–gene interaction network obtained as the union of all epistatic pairs detected either through physical or eQTL mapping. Fifteen out of the nineteen epistatic networks contain at least one hub, which we identify as a node of degree greater than expected by chance. Others [28, 29] have shown the role of such hubs in epistasis. In particular, nodes with high centrality can be observed on Fig. 2b (WASF2, FAK1) and Fig. 2a (GSK3-beta). Three of the remaining networks correspond, as for the maps where connectivity is not significant, to small maps with few selected edges (DM 3305, DM 4593, and DM 4794). Finally, in DM 4703, JNK3 (MAPK10), AKT (PKB) and Caspase-8 form the backbone of the epistatic network, but none of them accumulates sufficiently many connections to have significantly high centrality.

**Table 6:** Pairs of genes identified by eQTL mapping, filtered by direction of the epistatic effect, and involving a gene with known impact on multiple sclerosis

Map ID	Gene pair	Evidence
*Both genes have known impact on MS*		
5199	IP10 and NF-$\kappa$B	Both genes impact MS
*One gene only has known/suspected impact on MS*		
4455	alpha-V/beta-1 integrin and PCBP-1	alpha-V/beta-1 integrin probably impacts MS
4703	PADI2 and JNK1 (MAPK8)	PADI2 increases MS
4703	PADI2 and Caspase-3	
4703	PADI2 and Caspase-8	
5199	IP10 and IRF1	IP10 impacts MS
5199	IRF1 and NF-$\kappa$B	NF-$\kappa$B impacts MS
5199	JAK2 and PKA-reg (cAMP-dependent)	JAK2 impacts MS
5288	IL-1R1 and ROR-alpha	IL-1R1 probably impacts MS

#### Gene–gene epistatic interactions detected with EpiGWAS mostly complement known biological interactions

As can be observed on Fig. 2a, b, most of the gene–gene interactions we detected do not fall along edges already present in the original disease maps. On the one hand, the few interactions that are potentially very interesting, as they combine prior biological evidence with statistical evidence. Nonetheless, drawing conclusions about the underlying biology is challenging given the potential mismatch between biological epistasis and statistical epistasis [30].

On the other hand, this indicates that approaches that restrict themselves to testing for interactions along already known biological interactions [31], although facilitating interpretation, may miss out relevant interactions.

### 4 of the epistatic gene pairs involve missense variants

In total, we obtain 136 epistatic interactions based on physical mapping (see Table 2). As an exhaustive investigation of all these pairs is out of reach, we propose to focus on interactions where at least one of the SNPs can lead to a loss of function at the protein level. We are restricting ourselves here to interactions corresponding to a change of protein structure in at least one of the two genes, as they are the easiest to interpret. Other mechanisms are however possible, including splicing alterations, regulatory effects, or merely for the SNP to be in linkage disequilibrium with a missense variant.

The filtering yielded 4 gene pairs where one of the genes presents a variant reported as missense in BioMart [32] (Table 5). For each of those gene pairs, we also list the impact (activation, inhibition, or unspecified) on the MS phenotype, as given in the disease maps. Among these 4 pairs, the interaction between GLI-1 and SUFU is particularly interesting, since it matches a known biological interaction. Both genes are in direct binding interaction in DM 3305 (see Fig. 3), which describes Sonic Hedgehog signaling in oligodendrocyte precursor cells differentiation in MS. This map is described in more detail in Additional file 1: Additional file 1: Appendix C.2. In addition, GLI-1 is an important therapeutic target in cancer, including through its interaction with SUFU [33].

### Twenty-five of the epistatic gene pairs involve the up-regulation of both genes

Our pipeline selects a total of 112 epistatic interactions based on the eQTL mapping strategy (see Table 2). Again, as with physical mapping, additional filtering is needed to focus on a smaller number of pairs. Here we use the study of the direction of the epistatic effect (see “Direction of the epistatic effect” section) and select the gene pairs for which the coefficients $\beta _1, \beta _2, \beta _{{{\,\mathrm{syner}\,}}}$ share the same sign (that is to say, either all are positive, or all are negative). If all three coefficients are positive, inhibiting both genes reduces the risk for MS. On the contrary, if all three coefficients are negative, the two genes should be jointly activated to reduce MS risk.

This filtering leads to 25 gene pairs of interest across 13 maps (see Additional file 1: Table 1). We further list in Table 6 the pairs involving at least one gene with known or suspected effect on MS-related phenotypes, such as demyelination, remyelination failure, oligodendrocyte death, or damage of neural axons. This procedure highlights one specific pair, NF-$\kappa$B and IP10, where both genes are already known to have an impact on multiple sclerosis.

### NF-$\kappa$B and IP10 form a promising pair of interacting therapeutic targets for multiple sclerosis

In what follows, we further explore the potential of NF-$\kappa$B and IP10 as interacting therapeutic targets for multiple sclerosis. As stated in the previous section, the impact of both genes on the phenotype has already been specified, which justifies investigating their synergistic effect on the physiopathology of multiple sclerosis. Our analysis is focused on DM 5199 (see Fig. 4 and Additional file 1: Appendix C.4) where both genes belong to essential pathways.

Interferon-Inducible Cytokine IP10, also called CXCL10 (C-X-C motif chemokine ligand 10), is an antimicrobial gene which encodes a chemokine of the CXC subfamily, and is a ligand for the receptor CXCR3. This pro-inflammatory cytokine is involved in a wide variety of processes, including chemotaxis, differentiation, and activation of peripheral immune cells [34–36].

NF-$\kappa$B (nuclear factor kappa-light-chain-enhancer of activated B cells) is a protein complex involved in transcription, cell growth, and cytokine production. It plays a key role in the immune response to infection and has been linked to a number of diseases, including cancer, autoimmune disorders and sepsis.

NF-$\kappa$B upregulates the transcription of several genes, including IP10 [37]. Hence the two genes are in direct interaction on DM 5199. Figure 5a illustrates the impact of IP10 activation on T cells recruitment on the Central Nervous System. This allows intercellular contact between T cells and astrocytes presenting myelin antigens, which reactivates those T cells [38]. Reactivated T cells secrete pro-inflammatory cytokines; demyelination occurs and macrophages are activated. This further damages myelin and releases cytokines, which leads to the damage of neural axons [39] (see Fig. 5b).

There is currently no drug inhibiting IP10. However, two antibodies targeting IP10 are currently listed in ChEMBL [40] as having undergone clinical trials. The first one is NI-0801, which successfully passed Phase I for allergic contact dermatitis and Phase II for primary biliary cirrhosis. The second is Eldelumab (or MDX-100), which has completed Phase II trials for rheumatoid arthritis, Crohn’s disease and ulcerative colitis. While neither drug achieved its primary endpoint in clinical trials, they were both well-tolerated [41, 42], which is encouraging as to their potential use for other indications.

The inhibition of NF-$\kappa$B is a topic of major interest [43, 44]. The earliest example of FDA-approved inhibitor of NF-$\kappa$B is Bortezomib (also known as Velcade or PS-341) [45], indicated against multiple myeloma, mantle-cell lymphoma, and neoplasms. Most research on inhibiting NF-$\kappa$B focuses on its upstream regulators. For example, inhibitors of IKKB-beta (Inhibitor Of Nuclear Factor Kappa B Kinase Subunit Beta) aim at blocking the kinase which phosphorylates inhibitors of NF-kappa-B on two critical serine residues. Several small molecules antagonists targeting IKBKB have reached phase I, II and III clinical trials for several diseases [40].

Altogether, these clinical assays for IP10 and NF-$\kappa$B pathway inhibitors strengthen the potential of the pair as MS targets, where their simultaneous inhibition lowers the risk for MS.

## Conclusion

Targeted epistasis detection, which identifies interactions between a specific SNP and the rest of the genome, is an efficient way to reduce the statistical and computational burden of exhaustive pairwise testing. In addition, focusing on a single target SNP makes it possible to model its interactions with all other SNPs in the genome at once. However, the choice of a target SNP is not trivial. This SNP can for example be a top hit in a previous GWAS, or a SNP mapped to a gene that has an established biological relationship with the phenotype. In practice, one is likely to have a list of such target SNPs, rather than a single SNP to test. Targeted epistasis detection then yields a list of interacting SNPs (and their interaction scores) for each target SNP that is evaluated.

In this paper, we showed how to transform such an output into gene–gene interaction scores. This allows us to use targeted epistasis detection to obtain a list of pairs of genes in a given disease map that are likely to have a joint effect on disease risk. We illustrated our pipeline on multiple sclerosis, using a GWAS dataset from the Wellcome Trust Case Control Consortium, 19 multiple sclerosis disease maps from MetaCore, and EpiGWAS as a targeted epistasis detection tool.

The epistatic networks formed by the pairs of interacting genes we detected have several interesting topological properties: they form connected components; the epistatic networks obtained from physical and from eQTL mappings are complementary; they contain hubs; and they mostly complement known biological interactions from the disease maps.

Filtering the highest-scoring gene pairs allowed us to highlight two interactions as particularly promising in terms of therapeutic targeting in multiple sclerosis. The first one is the interaction between SUFU and GLI-1, which involves two potentially function-modifying variants. Although a more thorough investigation of the joint impact of these mutations on multiple sclerosis is warranted, GLI-1 is a therapeutic target of much interest in cancer, including through its interaction with SUFU, suggesting a starting point for further research.

The second one is the interaction between NF-$\kappa$B and IP10. Indeed, it corresponds to a well-characterized biological interaction. In addition, the analysis of the direction of their epistatic effect suggests that inhibiting both genes simultaneously has a negative impact on multiple sclerosis. Finally, several inhibitors of either genes have already been identified and passed Phase I clinical trial, suggesting promising drug candidates.

Identifying interactions between existing therapeutic targets directly allows for the development of combination therapies. Because of its focus on epistatic effects rather than independent effects, our pipeline can be of special interest in light of FDA guidance for the co-development of two or more drugs.^1^

Hence, an additional way to identify the most relevant interactions among those we select with EpiGWAS would be to explicitly look for pairs involving genes that are already therapeutic targets in other diseases. This could be achieved by crossing our epistatic networks with a data base such as OpenTargets [46].

Finally, hubs being the most influential nodes in a network, another potential strategy for therapeutic development would be to investigate whether the most central nodes of the epistatic networks could make good therapeutic targets. One example would be FAK1, with 12 interactions in DM 4901 (see Table 4).

## Supplementary Information

**Additional file 1**. This file compiles exhaustive results for all disease maps: network statistics, top scoring epistatic interactions and prioritised gene-gene pairs.

## Data Availability

The GWAS MS dataset is available by application to the Wellcome Trust Case Control Consortium Data Access Committee. The application process can be directly initiated from the following website https://www.wtccc.org.uk/info/access_to_data_samples.html. Access to data will be granted to all qualified investigators for appropriate use.

## Footnotes

1 Available for download from https://www.fda.gov/media/80100/download.
