# Supplementary material for: A systematic analysis of gene–gene interaction in multiple sclerosis
Source: BMC Med Genomics. 2022 Apr 30;15:100. doi: 10.1186/s12920-022-01247-3 (PMC9063218; doi:10.1186/s12920-022-01247-3)
Supplement: Supplementary file 1 — Additional file 1. This file compiles exhaustive results for all disease maps: network statistics, top scoring epistatic interactions and prioritised gene-gene pairs. [file 12920_2022_1247_MOESM1_ESM.pdf]

A systematic analysis of gene-gene interaction in multiple sclerosis
Supplementary Materials

March 8, 2022

A Network statistics

A.1 Size of the network

Size of the network built from epistatic pairs (positional mapping)

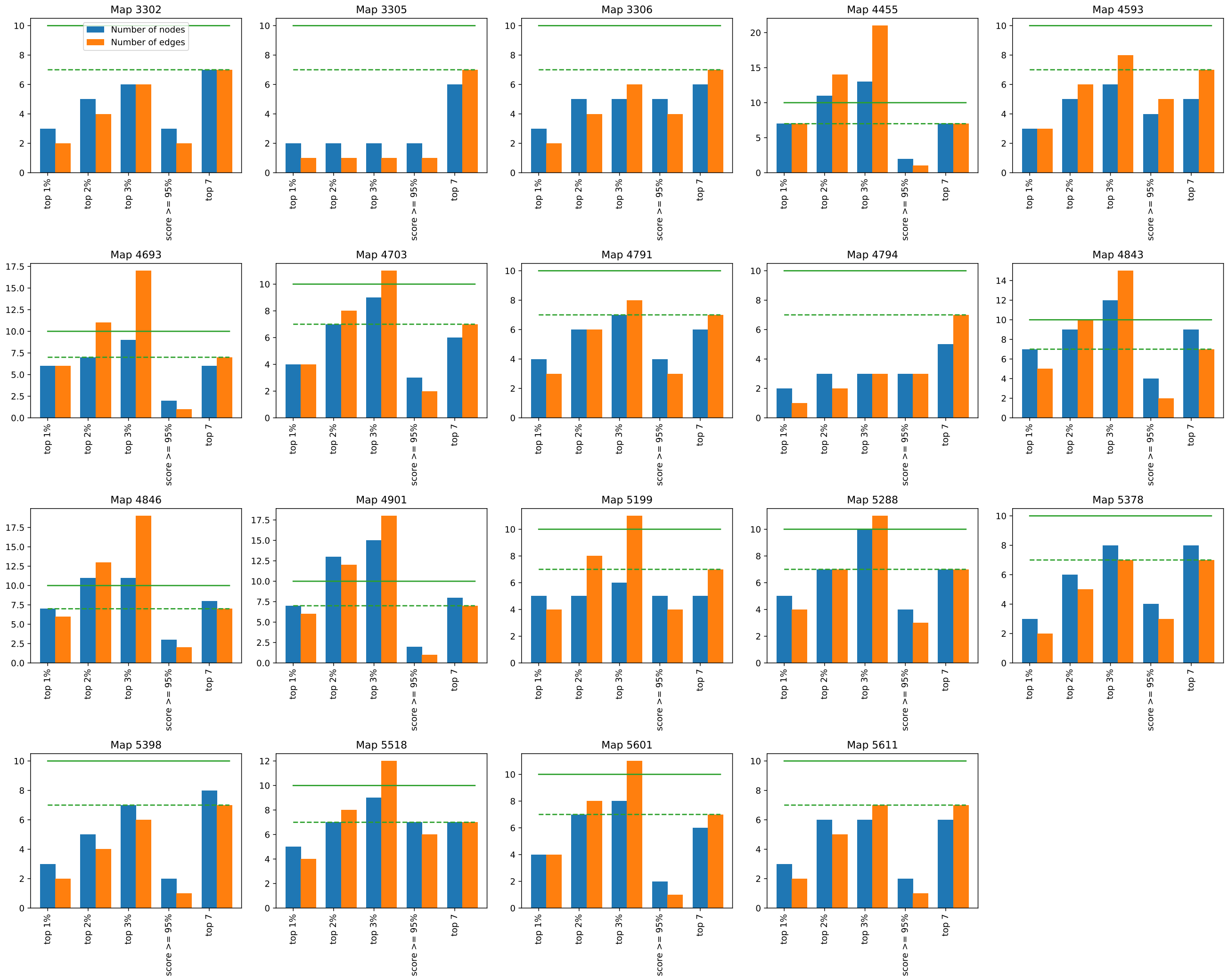

Size of the network built from epistatic pairs (eQTL mapping)

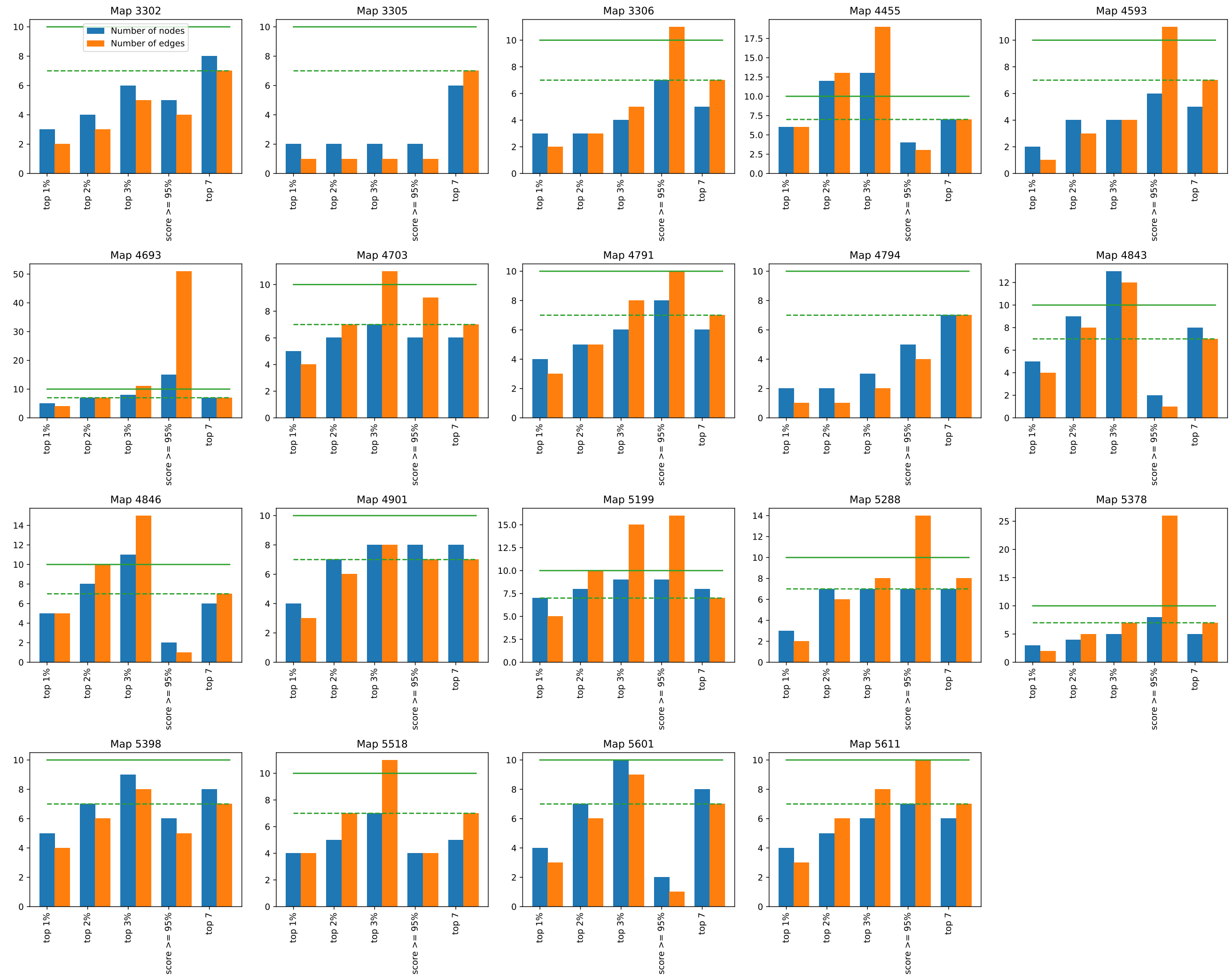

A.2 Connectivity

Yes means the epistatic interactions form a single connected component. p-value computed as the proportion of random networks (constructed by drawing the same number of edges over the same number of possible nodes) that also form a single connected component.

When picking the top 1% of interactions in each map

Map ID	Positional (p-value)	eQTL (p-value)	Joint (p-value)
3302	Yes (0.177)	Yes (0.201)	Yes (**0.032**)
3305	Yes (1.000)	Yes (1.000)	Yes (0.383)
3306	Yes (0.182)	Yes (0.203)	Yes (**0.027**)
4455	Yes (**0.001**)	Yes (**0.002**)	Yes (**0.000**)
4593	Yes (**0.049**)	Yes (1.000)	No (NA)
4693	Yes (**0.002**)	Yes (**0.017**)	Yes (**0.001**)
4703	Yes (**0.015**)	Yes (**0.017**)	Yes (**0.004**)
4791	Yes (**0.049**)	Yes (0.054)	Yes (**0.010**)
4794	Yes (1.000)	Yes (1.000)	No (NA)
4843	No (NA)	Yes (**0.014**)	No (NA)
4846	Yes (**0.002**)	Yes (**0.005**)	Yes (**0.001**)
4901	Yes (**0.002**)	Yes (0.052)	Yes (**0.002**)
5199	Yes (**0.016**)	No (NA)	Yes (**0.001**)
5288	Yes (**0.016**)	Yes (0.176)	Yes (**0.006**)
5378	Yes (0.180)	Yes (0.170)	No (NA)
5398	Yes (0.177)	Yes (**0.023**)	No (NA)
5518	Yes (**0.013**)	Yes (**0.015**)	No (NA)
5601	Yes (**0.015**)	Yes (**0.048**)	Yes (**0.004**)
5611	Yes (0.171)	Yes (0.051)	Yes (**0.015**)

When picking the top 2% of interactions in each map.

Map ID	Positional (p-value)	eQTL (p-value)	Joint (p-value)
3302	Yes (**0.035**)	Yes (0.077)	Yes (**0.020**)
3305	Yes (1.000)	Yes (1.000)	Yes (0.377)
3306	Yes (**0.034**)	Yes (0.078)	Yes (**0.016**)
4455	Yes (**0.000**)	Yes (**0.001**)	Yes (**0.018**)
4593	Yes (**0.009**)	Yes (0.092)	No (NA)
4693	Yes (**0.001**)	Yes (**0.006**)	Yes (**0.006**)
4703	Yes (**0.004**)	Yes (**0.005**)	Yes (**0.011**)
4791	Yes (**0.013**)	Yes (**0.018**)	Yes (**0.014**)
4794	Yes (0.256)	Yes (1.000)	Yes (0.098)
4843	No (NA)	Yes (**0.004**)	Yes (**0.009**)
4846	Yes (**0.001**)	Yes (**0.002**)	Yes (**0.017**)
4901	Yes (**0.001**)	Yes (**0.009**)	Yes (**0.004**)
5199	Yes (**0.003**)	Yes (**0.001**)	Yes (**0.011**)
5288	Yes (**0.005**)	Yes (**0.014**)	Yes (**0.012**)
5378	Yes (**0.018**)	Yes (**0.023**)	Yes (**0.018**)
5398	Yes (**0.032**)	Yes (**0.011**)	Yes (**0.014**)
5518	Yes (**0.004**)	Yes (**0.005**)	Yes (**0.011**)
5601	Yes (**0.004**)	Yes (**0.008**)	Yes (**0.013**)
5611	Yes (**0.018**)	Yes (**0.012**)	Yes (**0.015**)

When picking the top 3% of interactions in each map.

Map ID	Positional (p-value)	eQTL (p-value)	Joint (p-value)
3302	Yes (**0.019**)	Yes (**0.033**)	Yes (**0.042**)
3305	Yes (1.000)	Yes (1.000)	Yes (0.377)
3306	Yes (**0.017**)	Yes (**0.032**)	Yes (**0.029**)
4455	Yes (**0.003**)	Yes (**0.004**)	Yes (0.313)
4593	Yes (**0.009**)	Yes (0.060)	No (NA)
4693	Yes (**0.004**)	Yes (**0.006**)	Yes (0.107)
4703	Yes (**0.004**)	Yes (**0.008**)	Yes (0.100)
4791	Yes (**0.010**)	Yes (**0.014**)	Yes (0.064)
4794	Yes (0.117)	Yes (0.307)	Yes (0.055)
4843	No (NA)	Yes (**0.006**)	Yes (0.132)
4846	Yes (**0.004**)	Yes (**0.004**)	Yes (0.258)
4901	Yes (**0.004**)	Yes (**0.011**)	Yes (0.060)
5199	Yes (**0.005**)	Yes (**0.004**)	Yes (0.123)
5288	Yes (**0.007**)	Yes (**0.016**)	Yes (0.078)
5378	Yes (**0.014**)	Yes (**0.016**)	Yes (0.059)
5398	Yes (**0.019**)	Yes (**0.011**)	Yes (**0.039**)
5518	Yes (**0.004**)	Yes (**0.007**)	Yes (0.123)
5601	Yes (**0.005**)	Yes (**0.008**)	Yes (0.088)
5611	Yes (**0.015**)	Yes (**0.011**)	Yes (0.052)

When picking the interactions with a score greater than 95%

Map ID	Positional (p-value)	eQTL (p-value)	Joint (p-value)
3302	Yes (0.175)	Yes (**0.042**)	Yes (**0.020**)
3305	Yes (1.000)	Yes (1.000)	Yes (0.372)
3306	Yes (**0.032**)	Yes (0.082)	Yes (0.149)
4455	Yes (1.000)	Yes (**0.025**)	No (NA)
4593	Yes (**0.012**)	Yes (0.164)	No (NA)
4693	Yes (1.000)	Yes (0.985)	Yes (0.917)
4703	Yes (0.140)	Yes (**0.005**)	Yes (**0.005**)
4791	Yes (**0.050**)	Yes (**0.018**)	Yes (**0.033**)
4794	Yes (0.115)	Yes (0.155)	Yes (0.081)
4843	No (NA)	Yes (1.000)	No (NA)
4846	Yes (0.104)	Yes (1.000)	No (NA)
4901	Yes (1.000)	Yes (**0.011**)	Yes (**0.005**)
5199	Yes (**0.014**)	Yes (**0.005**)	Yes (**0.021**)
5288	Yes (**0.042**)	Yes (0.083)	Yes (0.104)
5378	Yes (0.059)	Yes (0.800)	Yes (0.862)
5398	Yes (1.000)	Yes (**0.015**)	No (NA)
5518	Yes (**0.004**)	Yes (**0.016**)	Yes (**0.004**)
5601	Yes (1.000)	Yes (1.000)	No (NA)
5611	Yes (1.000)	Yes (**0.012**)	Yes (**0.015**)

When picking the 7 top interactions

Map ID	Positional (p-value)	eQTL (p-value)	Joint (p-value)
3302	Yes (**0.018**)	Yes (**0.031**)	Yes (0.095)
3305	Yes (0.466)	Yes (0.822)	Yes (0.970)
3306	Yes (**0.017**)	Yes (**0.031**)	Yes (0.075)
4455	Yes (**0.001**)	Yes (**0.001**)	Yes (**0.000**)
4593	Yes (**0.009**)	Yes (0.053)	No (NA)
4693	Yes (**0.002**)	Yes (**0.006**)	Yes (**0.002**)
4703	Yes (**0.005**)	Yes (**0.005**)	Yes (**0.007**)
4791	Yes (**0.010**)	Yes (**0.013**)	Yes (**0.032**)
4794	Yes (0.095)	Yes (0.244)	Yes (0.444)
4843	No (NA)	Yes (**0.004**)	Yes (**0.003**)
4846	Yes (**0.001**)	Yes (**0.003**)	Yes (**0.002**)
4901	Yes (**0.001**)	Yes (**0.009**)	Yes (**0.003**)
5199	Yes (**0.005**)	Yes (**0.003**)	Yes (**0.002**)
5288	Yes (**0.006**)	Yes (**0.014**)	Yes (**0.026**)
5378	Yes (**0.018**)	Yes (**0.013**)	Yes (0.061)
5398	Yes (**0.019**)	Yes (**0.009**)	Yes (**0.040**)
5518	Yes (**0.004**)	Yes (**0.005**)	Yes (**0.008**)
5601	Yes (**0.005**)	Yes (**0.009**)	Yes (**0.013**)
5611	Yes (**0.015**)	Yes (**0.010**)	Yes (**0.040**)

A.3 Centrality

When picking the top 1% of interactions in each map

Map ID	positional		eQTL		joint	
	Max degree	Node(s) of max degree	Max degree	Node(s) of max degree	Max degree	Node(s) of max degree
3302	2 (0.183)	CNTN1 (F3)	2 (0.208)	ADAM17	3 (0.055)	ADAM17
3305	1 (1.000)	GLI-1, SUFU	1 (1.000)	IFN-gamma, SUFU	2 (0.359)	SUFU
3306	2 (0.184)	GSK3 beta	2 (0.193)	GSK3 beta	4 (**0.001**)	GSK3 beta
4455	6 (**0.000**)	FAK1	5 (**0.000**)	WASF2	6 (**0.000**)	FAK1
4593	2 (0.410)	AMPA receptor, GluR6, NCX1	1 (1.000)	Calpain 1(mu), NDUFA6	2 (0.672)	AMPA receptor, GluR6, NCX1
4693	5 (**0.000**)	mTORC1	3 (**0.037**)	mTORC1	8 (**0.000**)	mTORC1
4703	3 (**0.034**)	AKT(PKB)	3 (**0.038**)	Caspase-8	4 (**0.028**)	AKT(PKB)
4791	3 (**0.013**)	AKT(PKB)	3 (**0.013**)	PI3K reg class IA	3 (0.185)	AKT(PKB), PI3K reg class IA
4794	1 (1.000)	DHA2, PDGF-R-alpha	1 (1.000)	GALC, RXRA	1 (1.000)	DHA2, GALC, PDGF-R-alpha, RXRA
4843	4 (**0.003**)	AKT(PKB)	4 (**0.000**)	SHP-2	4 (**0.035**)	AKT(PKB), SHP-2
4846	6 (**0.000**)	Neuregulin 1	4 (**0.001**)	Bcl-XL	7 (**0.000**)	Neuregulin 1
4901	6 (**0.000**)	FAK1	3 (**0.013**)	HYAL3	6 (**0.000**)	FAK1
5199	4 (**0.000**)	PKA-cat (cAMP-dependent)	3 (0.067)	JAK2	4 (**0.042**)	JAK2, PKA-cat (cAMP-dependent)
5288	4 (**0.001**)	ROR-alpha	2 (0.172)	IL-1RI	5 (**0.000**)	ROR-alpha
5378	2 (0.176)	JNK(MAPK8-10)	2 (0.172)	NF-kB	2 (0.693)	JNK(MAPK8-10), NF-kB
5398	2 (0.186)	AKT(PKB)	4 (**0.001**)	TRADD	4 (**0.011**)	TRADD
5518	4 (**0.000**)	AKT(PKB)	3 (**0.037**)	PI3K reg class IA	4 (**0.028**)	AKT(PKB)
5601	3 (**0.035**)	AKT(PKB)	3 (**0.010**)	Bcl-XL	3 (0.244)	AKT(PKB), Bcl-XL, GSK3 beta
5611	2 (0.177)	Granzyme B	2 (0.433)	IFN-gamma, KLRK1 (NKG2D)	3 (0.094)	KLRK1 (NKG2D)

When picking the top 2% of interactions in each map

Map ID	positional		eQTL		joint	
	Max degree	Node(s) of max degree	Max degree	Node(s) of max degree	Max degree	Node(s) of max degree
3302	4 (**0.001**)	CNTN1 (F3)	3 (**0.019**)	ADAM17	4 (**0.033**)	ADAM17, CNTN1 (F3)
3305	1 (1.000)	GLI-1, SUFU	1 (1.000)	IFN-gamma, SUFU	2 (0.365)	SUFU
3306	4 (**0.002**)	GSK3 beta	2 (0.499)	APC protein, Beta-catenin, GSK3 beta	6 (**0.000**)	GSK3 beta
4455	10 (**0.000**)	FAK1	10 (**0.000**)	WASF2	11 (**0.000**)	WASF2
4593	4 (**0.013**)	NCX1	2 (0.549)	Calpain 1(mu), NDUFA6	4 (0.086)	NCX1
4693	6 (**0.001**)	mTORC1	4 (**0.018**)	mTORC1	10 (**0.000**)	mTORC1
4703	4 (**0.033**)	AKT(PKB), JNK3(MAPK10)	5 (**0.001**)	Caspase-8	5 (0.056)	AKT(PKB), Caspase-8
4791	5 (**0.000**)	AKT(PKB)	4 (**0.005**)	PI3K reg class IA	5 (**0.018**)	AKT(PKB), PI3K reg class IA
4794	2 (0.241)	DHA2	1 (1.000)	GALC, RXRA	2 (0.553)	DHA2, GALC
4843	5 (**0.005**)	AKT(PKB)	8 (**0.000**)	SHP-2	8 (**0.000**)	SHP-2
4846	9 (**0.000**)	Neuregulin 1	6 (**0.000**)	ErbB2	11 (**0.000**)	Neuregulin 1
4901	12 (**0.000**)	FAK1	6 (**0.000**)	HYAL3	12 (**0.000**)	FAK1
5199	4 (**0.032**)	JAK1, PKA-cat (cAMP-dependent)	4 (0.064)	IRF1, JAK2	7 (**0.001**)	JAK2
5288	6 (**0.000**)	ROR-alpha	6 (**0.000**)	IL-1RI	6 (**0.001**)	IL-1RI, ROR-alpha
5378	5 (**0.000**)	JNK(MAPK8-10)	3 (0.135)	VCAM1, sVCAM1	5 (**0.013**)	JNK(MAPK8-10)
5398	4 (**0.001**)	AKT(PKB)	6 (**0.000**)	TRADD	6 (**0.001**)	TRADD
5518	6 (**0.000**)	AKT(PKB)	4 (**0.019**)	IL-18R1, PI3K reg class IA	6 (**0.004**)	AKT(PKB)
5601	5 (**0.001**)	AKT(PKB)	6 (**0.000**)	Bcl-XL	7 (**0.000**)	Bcl-XL
5611	5 (**0.000**)	Granzyme B	4 (**0.013**)	KLRK1 (NKG2D)	5 (**0.018**)	Granzyme B, KLRK1 (NKG2D)

When picking the top 3% of interactions in each map

Map ID	positional		eQTL		joint	
	Max degree	Node(s) of max degree	Max degree	Node(s) of max degree	Max degree	Node(s) of max degree
3302	5 (**0.000**)	CNTN1 (F3)	5 (**0.000**)	ADAM17	7 (**0.000**)	ADAM17
3305	1 (1.000)	GLI-1, SUFU	1 (1.000)	IFN-gamma, SUFU	2 (0.369)	SUFU
3306	4 (**0.020**)	GSK3 beta	3 (0.158)	Beta-catenin, GSK3 beta	7 (**0.000**)	GSK3 beta
4455	12 (**0.000**)	FAK1	11 (**0.000**)	WASF2	12 (**0.000**)	FAK1, WASF2
4593	5 (**0.003**)	NCX1	3 (0.089)	NDUFA6	5 (**0.032**)	NCX1
4693	8 (**0.000**)	mTORC1	5 (**0.015**)	mTORC1	12 (**0.000**)	mTORC1
4703	7 (**0.000**)	JNK3(MAPK10)	6 (**0.001**)	Caspase-8	7 (**0.006**)	JNK3(MAPK10)
4791	6 (**0.000**)	AKT(PKB)	5 (**0.004**)	PI3K reg class IA	6 (**0.015**)	AKT(PKB), PI3K reg class IA
4794	2 (0.593)	DHA2, GALC, PDGF-R-alpha	2 (0.305)	GALC	4 (**0.011**)	GALC
4843	8 (**0.000**)	AKT(PKB)	12 (**0.000**)	SHP-2	12 (**0.000**)	SHP-2
4846	9 (**0.000**)	Neuregulin 1	8 (**0.000**)	ErbB2	11 (**0.000**)	Neuregulin 1
4901	14 (**0.000**)	FAK1	7 (**0.000**)	HYAL3	14 (**0.000**)	FAK1
5199	5 (**0.014**)	JAK2	5 (**0.040**)	IP10, IRF1, JAK2	9 (**0.000**)	JAK2
5288	9 (**0.000**)	ROR-alpha	6 (**0.000**)	IL-1RI	9 (**0.000**)	ROR-alpha
5378	7 (**0.000**)	JNK(MAPK8-10)	4 (**0.035**)	VCAM1, sVCAM1	7 (**0.001**)	JNK(MAPK8-10)
5398	6 (**0.000**)	AKT(PKB)	8 (**0.000**)	TRADD	8 (**0.000**)	TRADD
5518	8 (**0.000**)	AKT(PKB)	6 (**0.001**)	IL-18R1, PI3K reg class IA	8 (**0.001**)	AKT(PKB)
5601	6 (**0.001**)	AKT(PKB), FOXO3A	9 (**0.000**)	Bcl-XL	11 (**0.000**)	Bcl-XL
5611	5 (**0.001**)	Granzyme B	5 (**0.003**)	KLRK1 (NKG2D)	7 (**0.001**)	KLRK1 (NKG2D)

When picking the interactions with a score greater than 95%

Map ID	positional		eQTL		joint	
	Max degree	Node(s) of max degree	Max degree	Node(s) of max degree	Max degree	Node(s) of max degree
3302	2 (0.179)	CNTN1 (F3)	4 (**0.002**)	ADAM17	5 (**0.000**)	ADAM17
3305	1 (1.000)	GLI-1, SUFU	1 (1.000)	IFN-gamma, SUFU	2 (0.364)	SUFU
3306	4 (**0.001**)	GSK3 beta	6 (**0.004**)	Beta-catenin	8 (**0.000**)	GSK3 beta
4455	1 (1.000)	FAK1, Fyn	3 (**0.006**)	WASF2	3 (**0.018**)	WASF2
4593	3 (0.111)	AMPA receptor, NCX1	5 (0.059)	Calpain 1(mu), NDUFA6	5 (0.143)	Calpain 1(mu), NDUFA6
4693	1 (1.000)	AKT(PKB), mTORC1	13 (**0.000**)	mTORC1	13 (**0.000**)	mTORC1
4703	2 (0.134)	AKT(PKB)	5 (**0.004**)	Caspase-8	5 (**0.010**)	Caspase-8
4791	3 (**0.013**)	AKT(PKB)	6 (**0.001**)	PI3K reg class IA	6 (**0.005**)	PI3K reg class IA
4794	2 (0.599)	DHA2, GALC, PDGF-R-alpha	4 (**0.005**)	GALC	6 (**0.000**)	GALC
4843	1 (1.000)	AKT(PKB), GRB2, PI3K cat class IA, c-Raf-1	1 (1.000)	PI3K reg class IA, SHP-2	1 (1.000)	AKT(PKB), GRB2, PI3K cat class IA, I3K reg class IA, SHP-2, c-Raf-1
4846	2 (0.108)	Neuregulin 1	1 (1.000)	Bcl-XL, ErbB2	2 (0.287)	Neuregulin 1
4901	1 (1.000)	FAK1, Fyn	7 (**0.000**)	HYAL3	7 (**0.000**)	HYAL3
5199	4 (**0.001**)	PKA-cat (cAMP-dependent)	5 (0.057)	IP10, IRF1, JAK2, PPAR-gamma	6 (**0.022**)	JAK2, PPAR-gamma
5288	3 (**0.009**)	ROR-alpha	6 (**0.010**)	IL-1RI	7 (**0.001**)	ROR-alpha
5378	3 (**0.015**)	JNK(MAPK8-10)	7 (0.087)	5'-NTD, ITGA4, NF-kB, VCAM1, sVCAM1	8 (**0.026**)	ITGA4
5398	1 (1.000)	AKT(PKB), IFN-alpha/beta receptor	5 (**0.000**)	TRADD	5 (**0.000**)	TRADD
5518	6 (**0.000**)	AKT(PKB)	3 (**0.036**)	PI3K reg class IA	6 (**0.000**)	AKT(PKB)
5601	1 (1.000)	AKT(PKB), FOXO3A	1 (1.000)	Bcl-XL, Rb protein	1 (1.000)	AKT(PKB), Bcl-XL, FOXO3A, Rb protein
5611	1 (1.000)	Granzyme B, KLRK1 (NKG2D)	6 (**0.000**)	KLRK1 (NKG2D)	6 (**0.000**)	KLRK1 (NKG2D)

When picking the 7 top interactions

Map ID	positional		eQTL		joint	
	Max degree	Node(s) of max degree	Max degree	Node(s) of max degree	Max degree	Node(s) of max degree
3302	6 (**0.000**)	CNTN1 (F3)	7 (**0.000**)	ADAM17	9 (**0.000**)	ADAM17
3305	5 (**0.021**)	GLI-2	5 (**0.032**)	SUFU	7 (**0.013**)	SUFU
3306	5 (**0.002**)	GSK3 beta	4 (**0.049**)	GSK3 beta	7 (**0.000**)	GSK3 beta
4455	6 (**0.000**)	FAK1	6 (**0.000**)	WASF2	6 (**0.001**)	FAK1, WASF2
4593	4 (**0.027**)	AMPA receptor, NCX1	4 (0.063)	Calpain 1(mu), NDUFA6	4 (0.431)	AMPA receptor, Calpain 1(mu), NCX1, NDUFA6
4693	5 (**0.000**)	mTORC1	4 (**0.021**)	mTORC1	9 (**0.000**)	mTORC1
4703	4 (**0.016**)	AKT(PKB)	5 (**0.001**)	Caspase-8	5 (**0.036**)	AKT(PKB), Caspase-8
4791	5 (**0.002**)	AKT(PKB)	4 (**0.030**)	PI3K reg class IA	5 (0.068)	AKT(PKB), PI3K reg class IA
4794	4 (0.082)	GALC, PDGF-R-alpha	6 (**0.000**)	GALC	8 (**0.000**)	GALC
4843	5 (**0.000**)	AKT(PKB)	7 (**0.000**)	SHP-2	7 (**0.000**)	SHP-2
4846	7 (**0.000**)	Neuregulin 1	5 (**0.000**)	Bcl-XL	9 (**0.000**)	Neuregulin 1
4901	7 (**0.000**)	FAK1	7 (**0.000**)	HYAL3	7 (**0.000**)	FAK1, HYAL3
5199	4 (**0.017**)	PKA-cat (cAMP-dependent)	4 (**0.013**)	JAK2	7 (**0.000**)	JAK2
5288	6 (**0.000**)	ROR-alpha	6 (**0.000**)	IL-1RI	6 (**0.004**)	IL-1RI, ROR-alpha
5378	7 (**0.000**)	JNK(MAPK8-10)	4 (**0.033**)	VCAM1, sVCAM1	7 (**0.001**)	JNK(MAPK8-10)
5398	7 (**0.000**)	AKT(PKB)	7 (**0.000**)	TRADD	7 (**0.001**)	AKT(PKB), TRADD
5518	6 (**0.000**)	AKT(PKB)	4 (**0.023**)	IL-18R1, PI3K reg class IA	6 (**0.003**)	AKT(PKB)
5601	4 (**0.017**)	AKT(PKB), FOXO3A	7 (**0.000**)	Bcl-XL	7 (**0.000**)	Bcl-XL
5611	5 (**0.002**)	Granzyme B	5 (**0.001**)	KLRK1 (NKG2D)	7 (**0.001**)	KLRK1 (NKG2D)

B Visualization of epiGWAS results on MetaCore disease maps

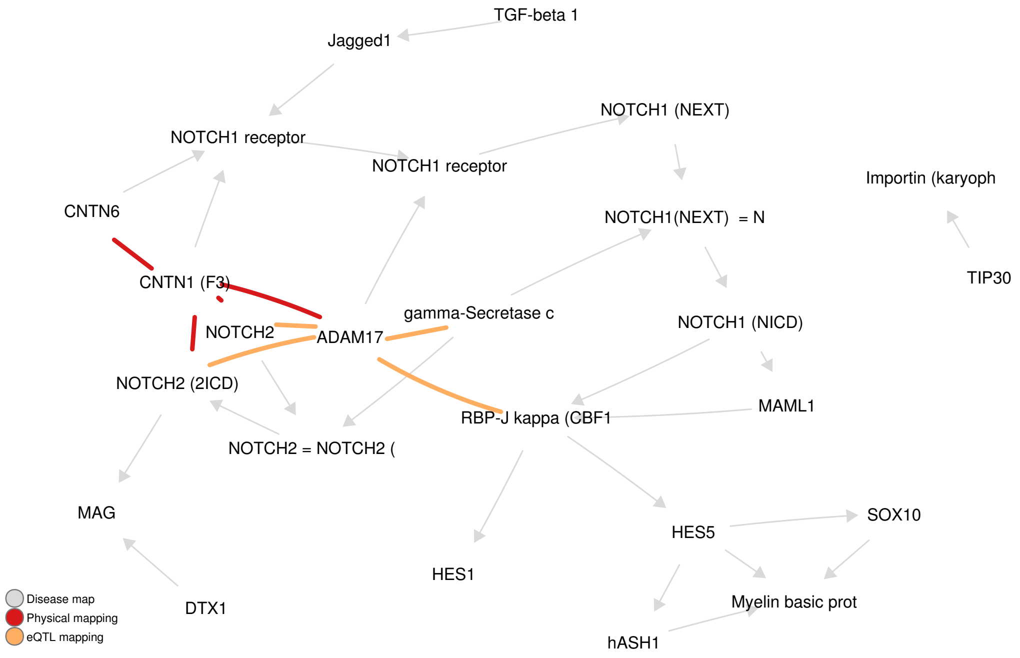

(a) DM 3302: Notch signaling in oligodendrocyte precursor cell differentiation in multiple sclerosis

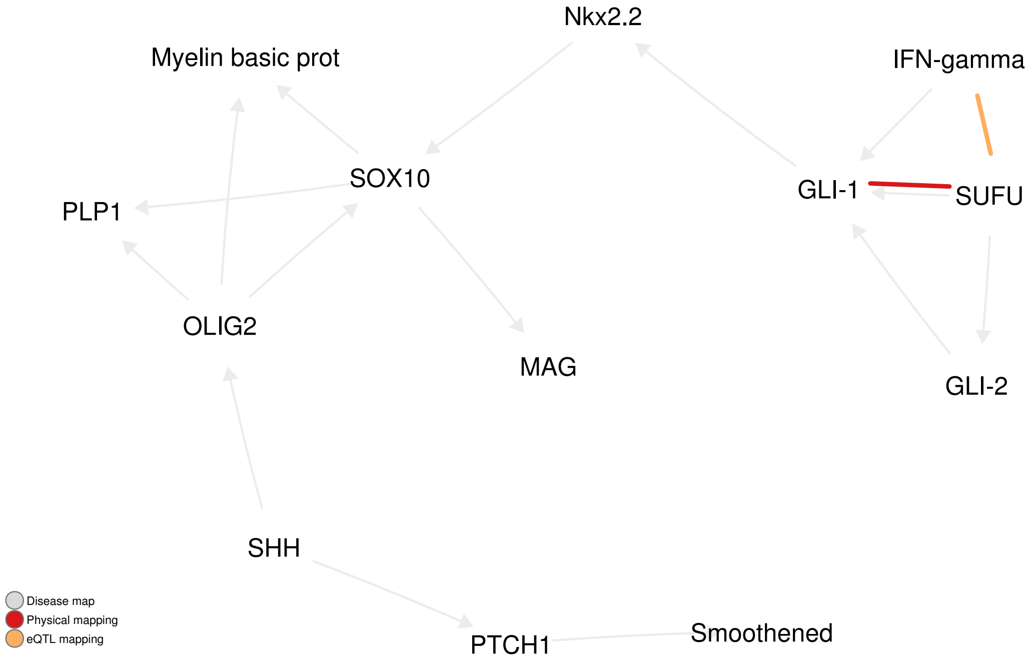

(b) DM 3305: SHH signaling in oligodendrocyte precursor cells differentiation in multiple sclerosis

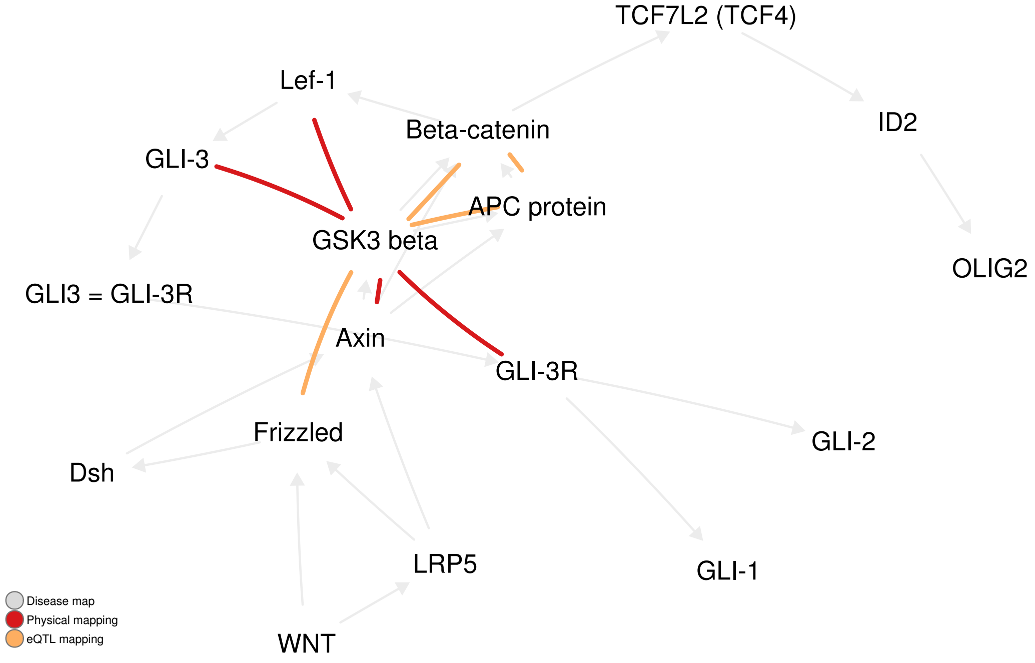

(c) DM 3306: Inhibition of oligodendrocyte precursor cells differentiation by Wnt signaling in multiple sclerosis

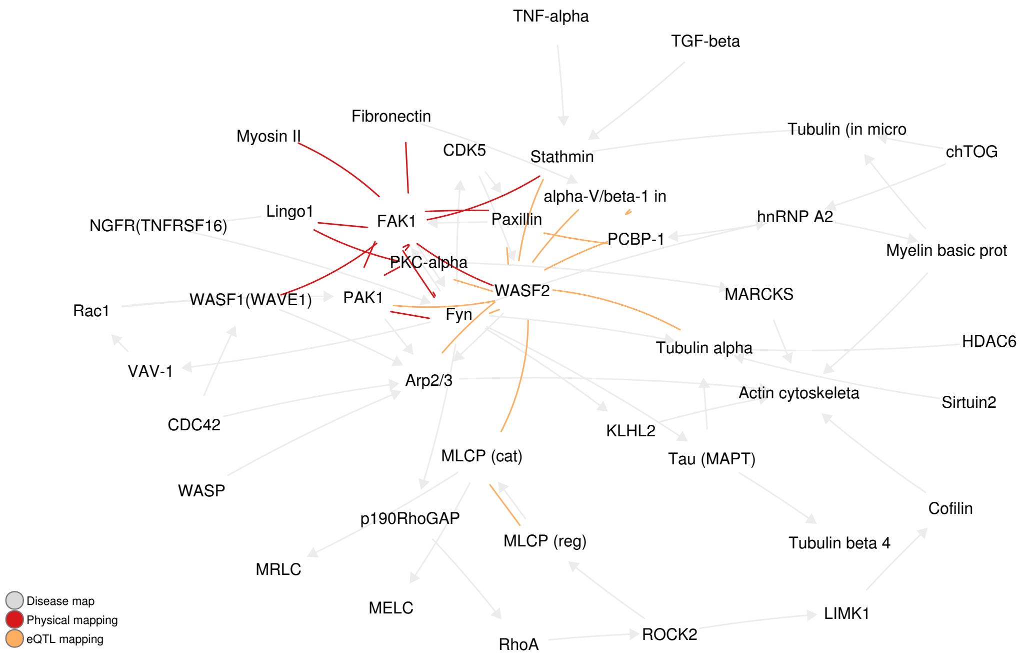

(d) DM 4455: Inhibition of remyelination in multiple sclerosis: regulation of cytoskeleton proteins

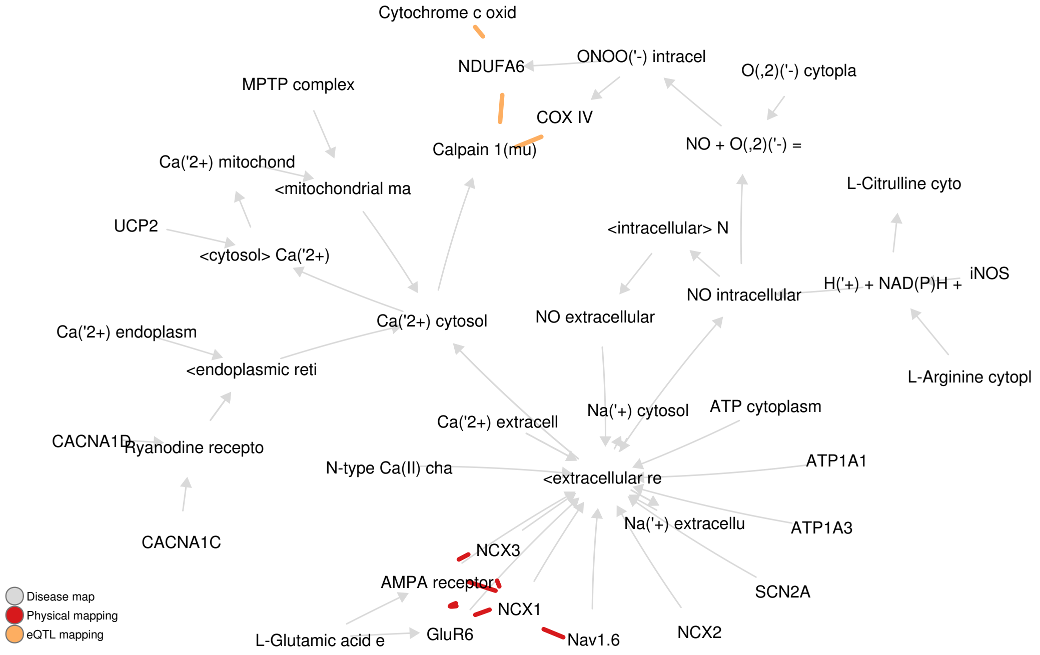

(e) DM 4593: Axonal degeneration in multiple sclerosis

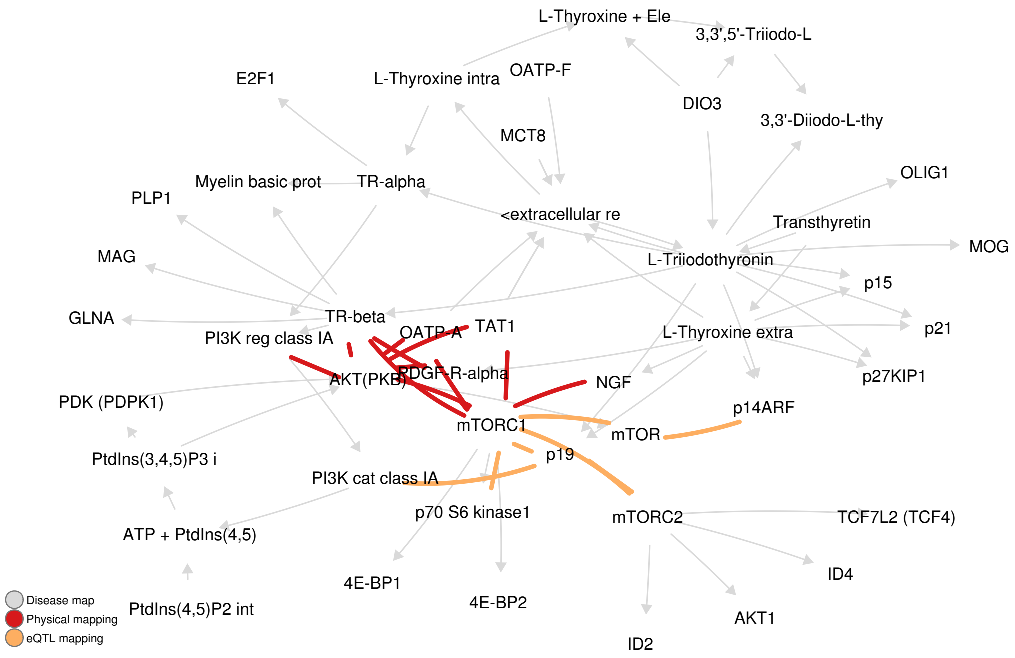

(f) DM 4693: Role of Thyroid hormone in regulation of oligodendrocyte differentiation in multiple sclerosis

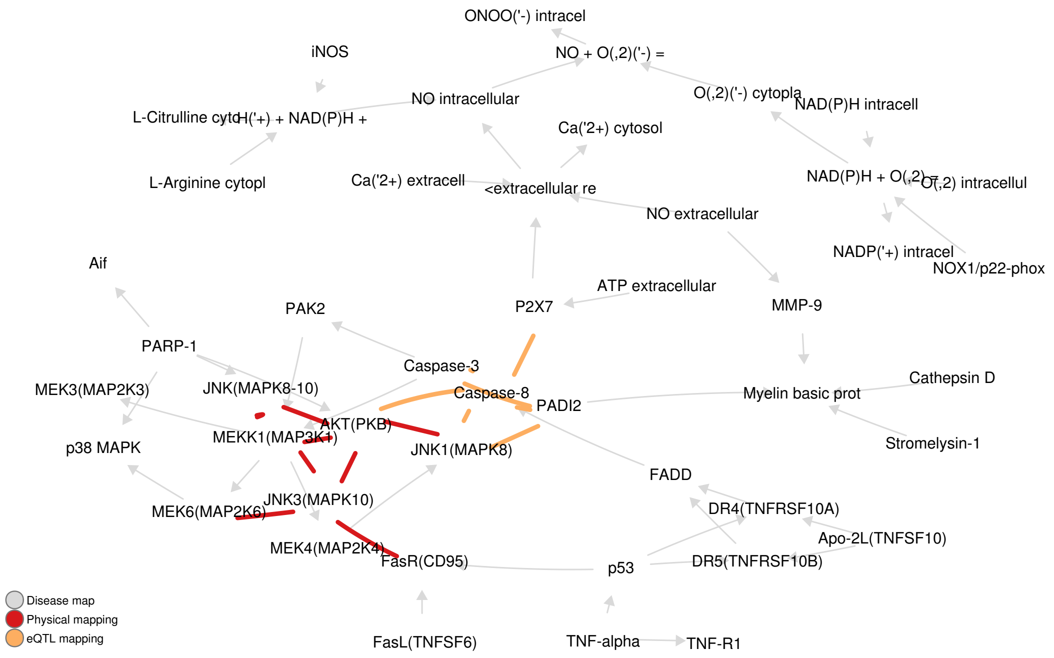

(g) DM 4703: Demyelination in multiple sclerosis

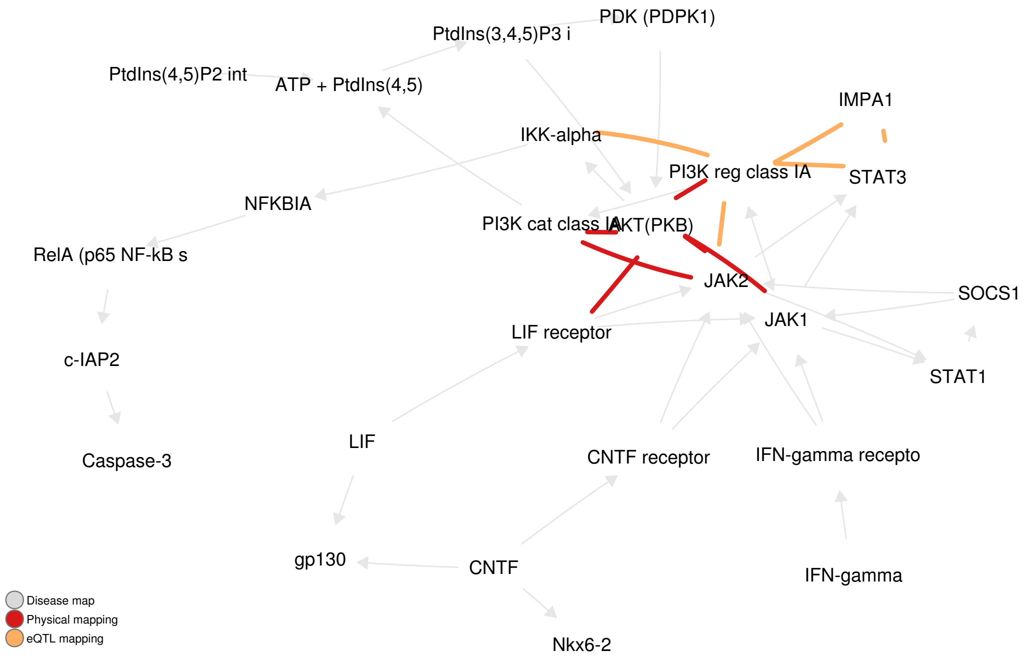

(h) DM 4791: Role of CNTF and LIF in regulation of oligodendrocyte development in multiple sclerosis

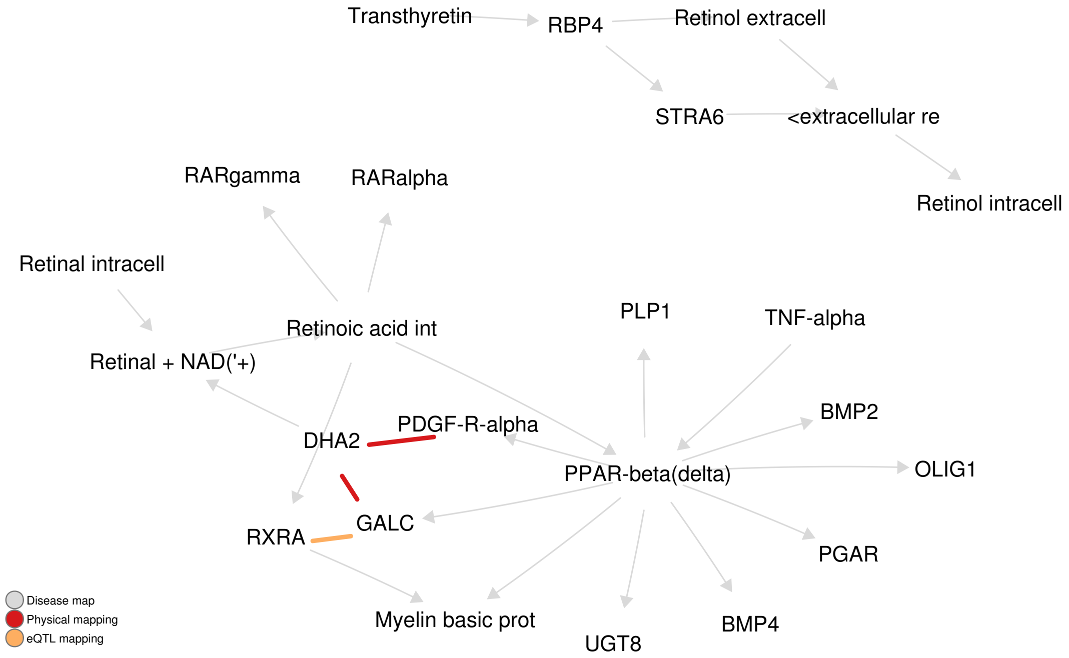

(i) DM 4794: Retinoic acid regulation of oligodendrocyte differentiation in multiple sclerosis

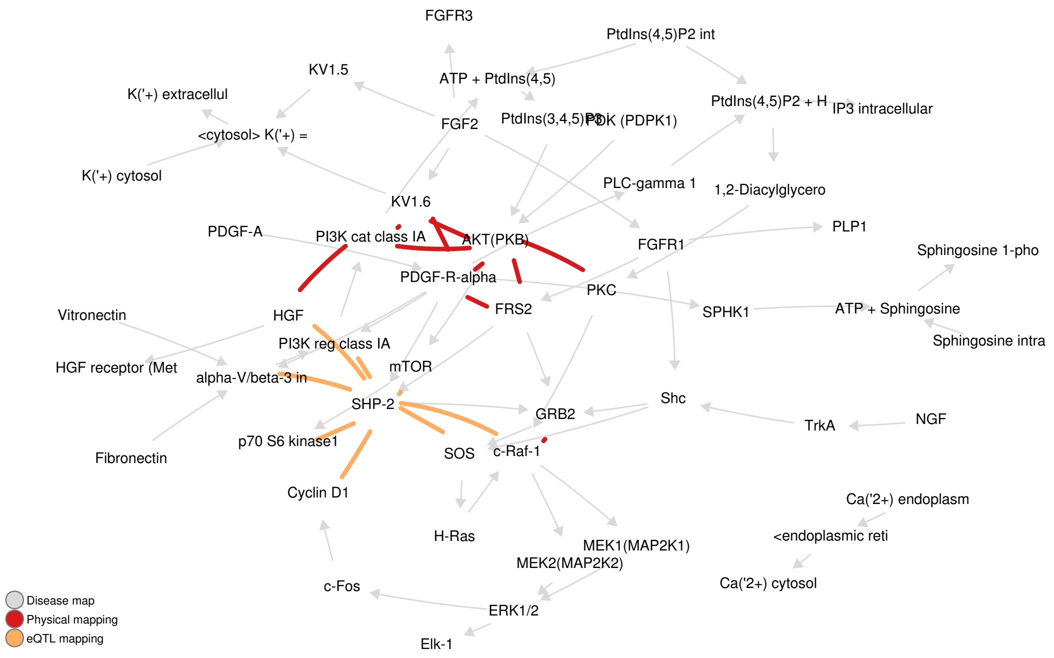

(j) DM 4843: Growth factors in regulation of oligodendrocyte precursor cells proliferation in multiple sclerosis

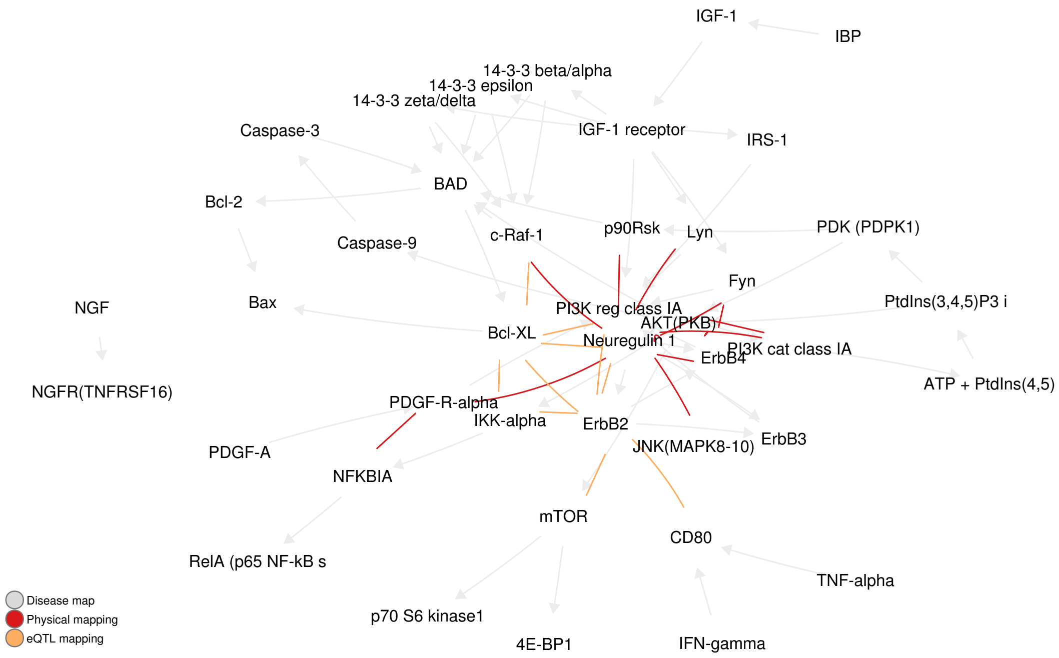

(k) DM 4846: Growth factors in regulation of oligodendrocyte precursor cells survival in multiple sclerosis

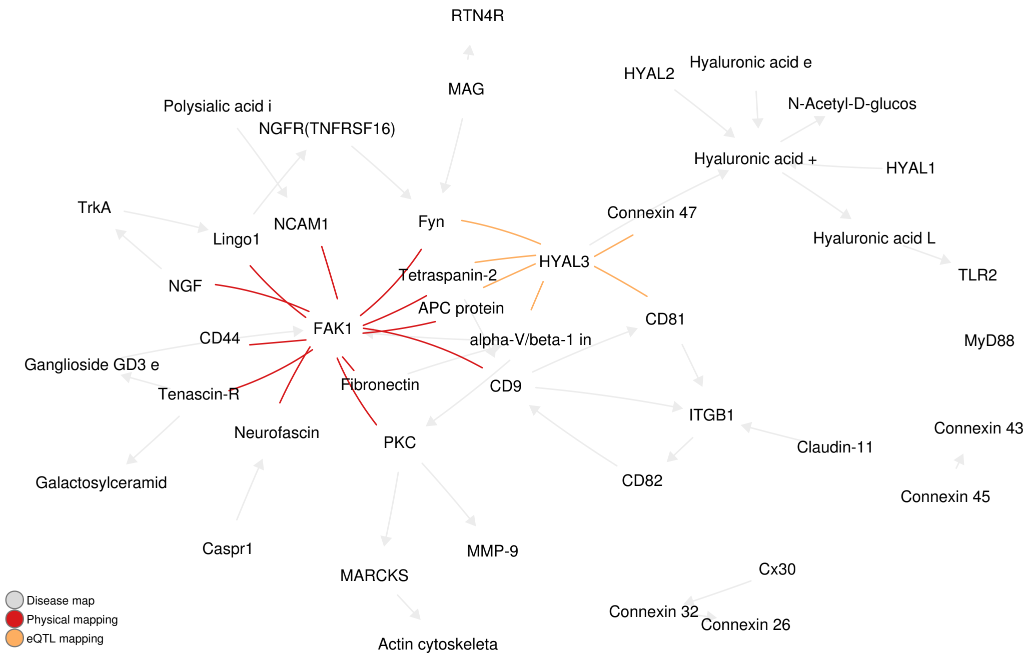

(l) DM 4901: Inhibition of remyelination in multiple sclerosis: role of cell-cell and ECM-cell interactions

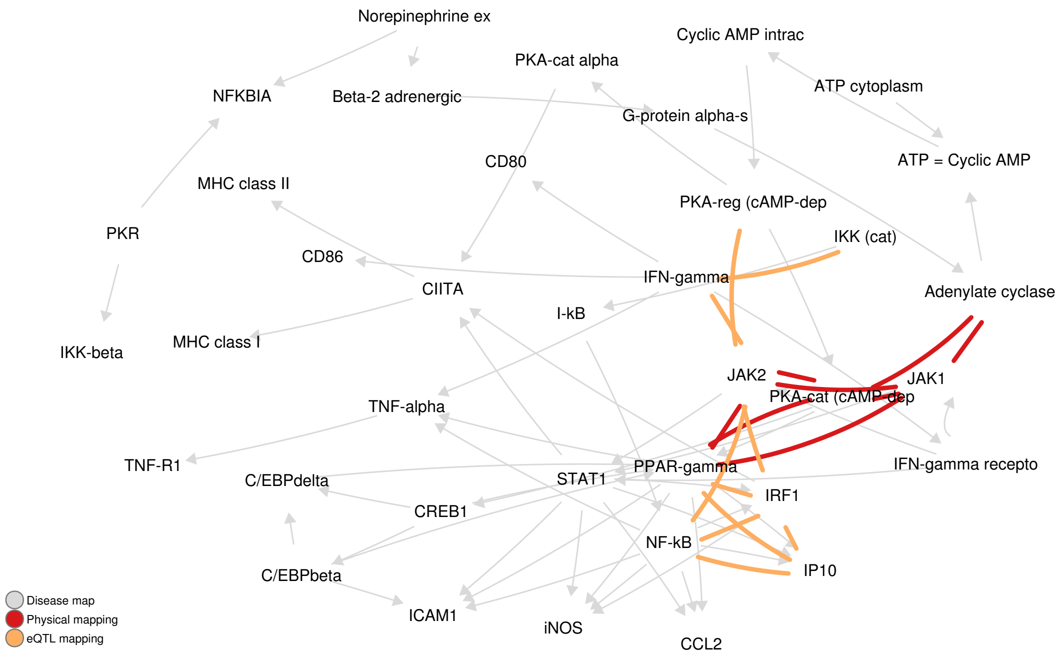

(m) DM 5199: Cooperative action of IFN-γ and TNF-α on astrocytes in multiple sclerosis

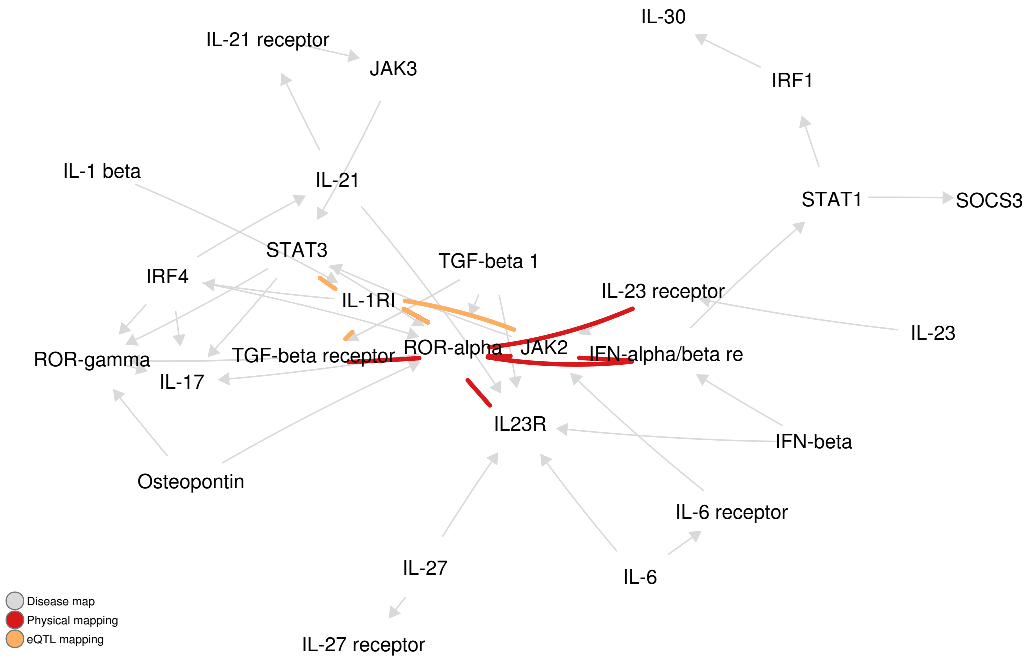

(n) DM 5288: Impaired inhibition of Th17 cell differentiation by IFN-β in multiple sclerosis

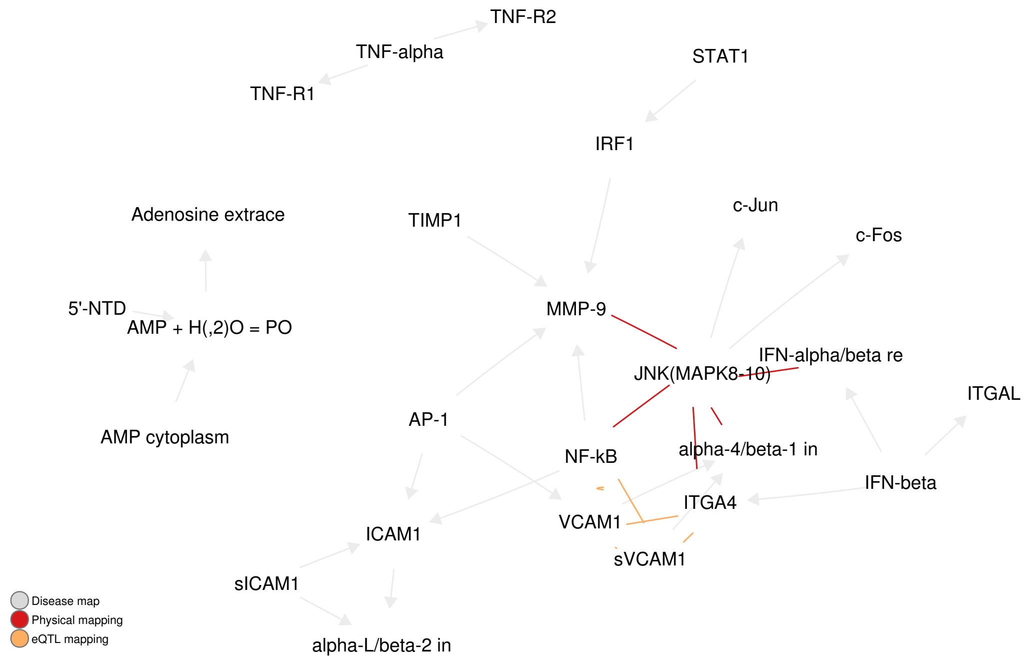

(o) DM 5378: Role of IFN-β in the improvement of blood-brain barrier integrity in multiple sclerosis

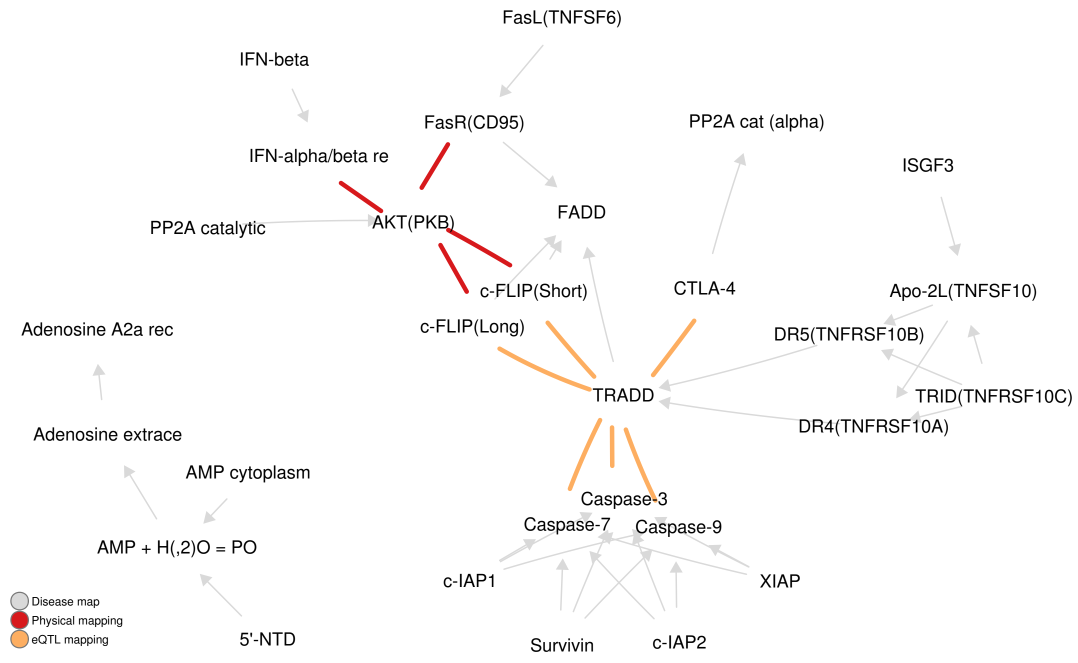

(p) DM 5398: Role of IFN-β in activation of T cell apoptosis in multiple sclerosis

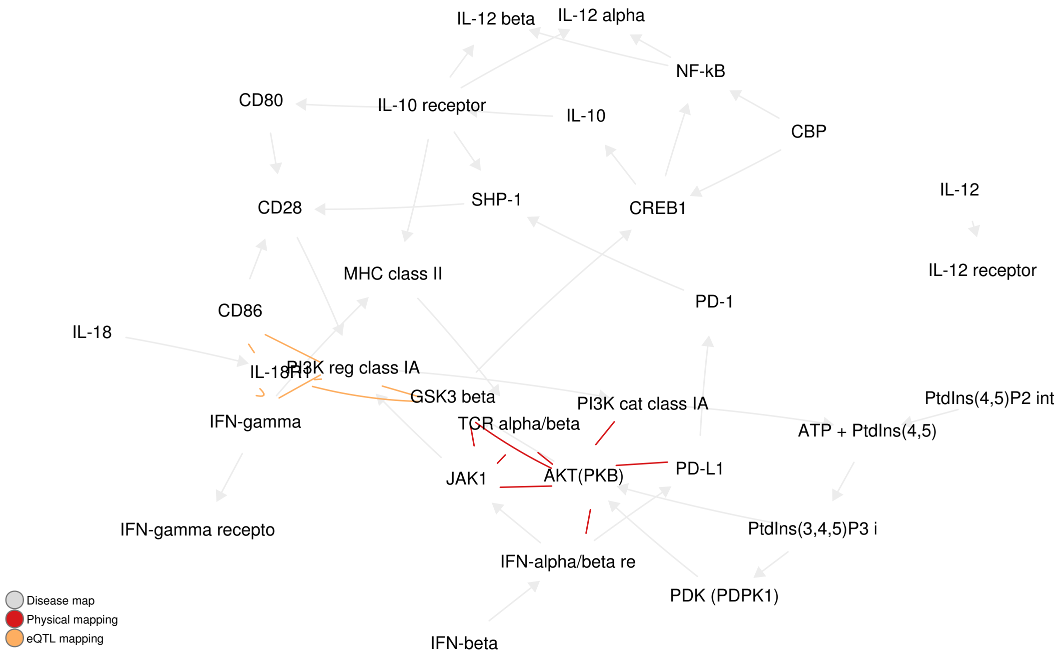

(q) DM 5518: Role of IFN-β in inhibition of Th1 cell differentiation in multiple sclerosis

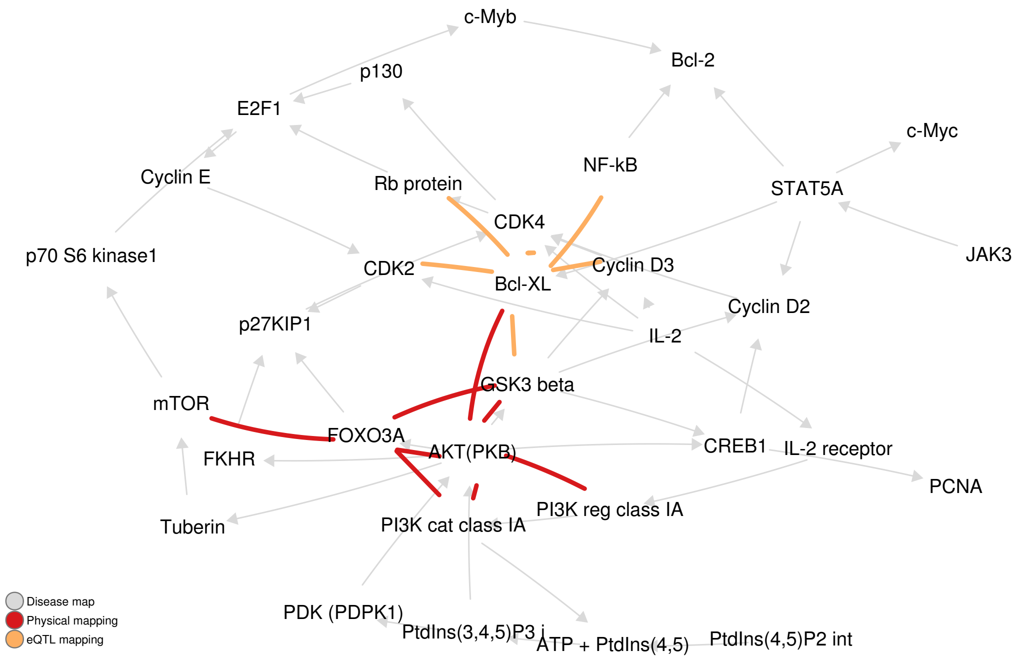

(r) DM 5601: IL-2 as a growth factor for T cells in multiple sclerosis

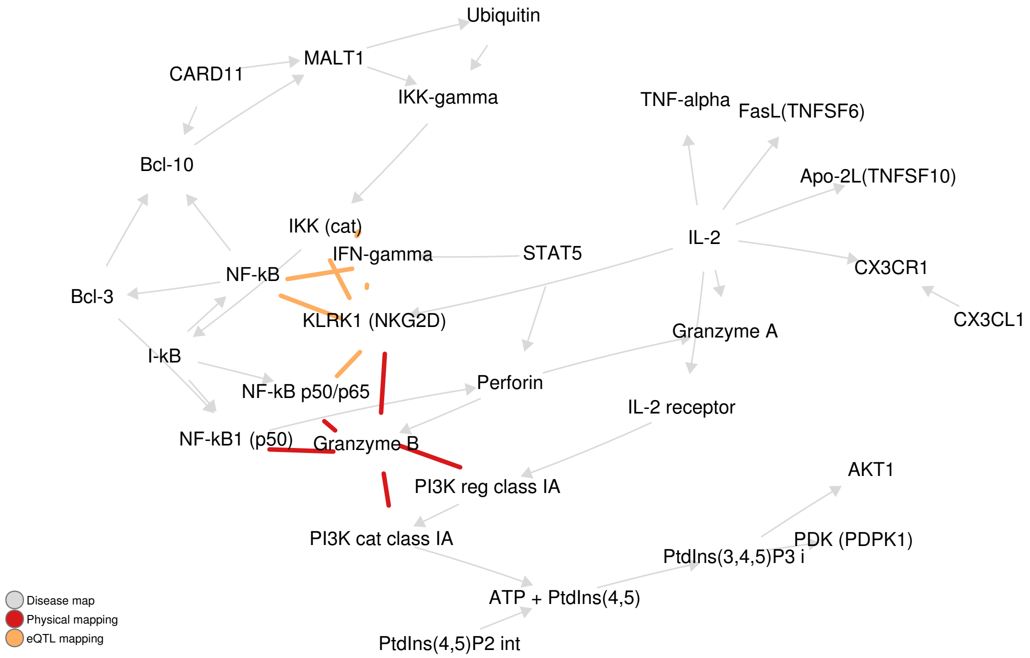

(s) DM 5611: Role of IL-2 in the enhancement of NK cell cytotoxicity in multiple sclerosis

Figure 1: Representation of the 2% top-scoring interactions for physical and eQTL mappings on the original disease maps.

C MetaCore disease maps

C.1 The different types of links between proteins/proteins or proteins-phenotypes in MetaCore maps

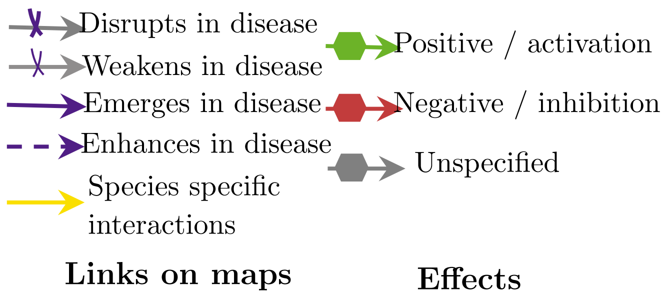

C.2 Disease map 3305

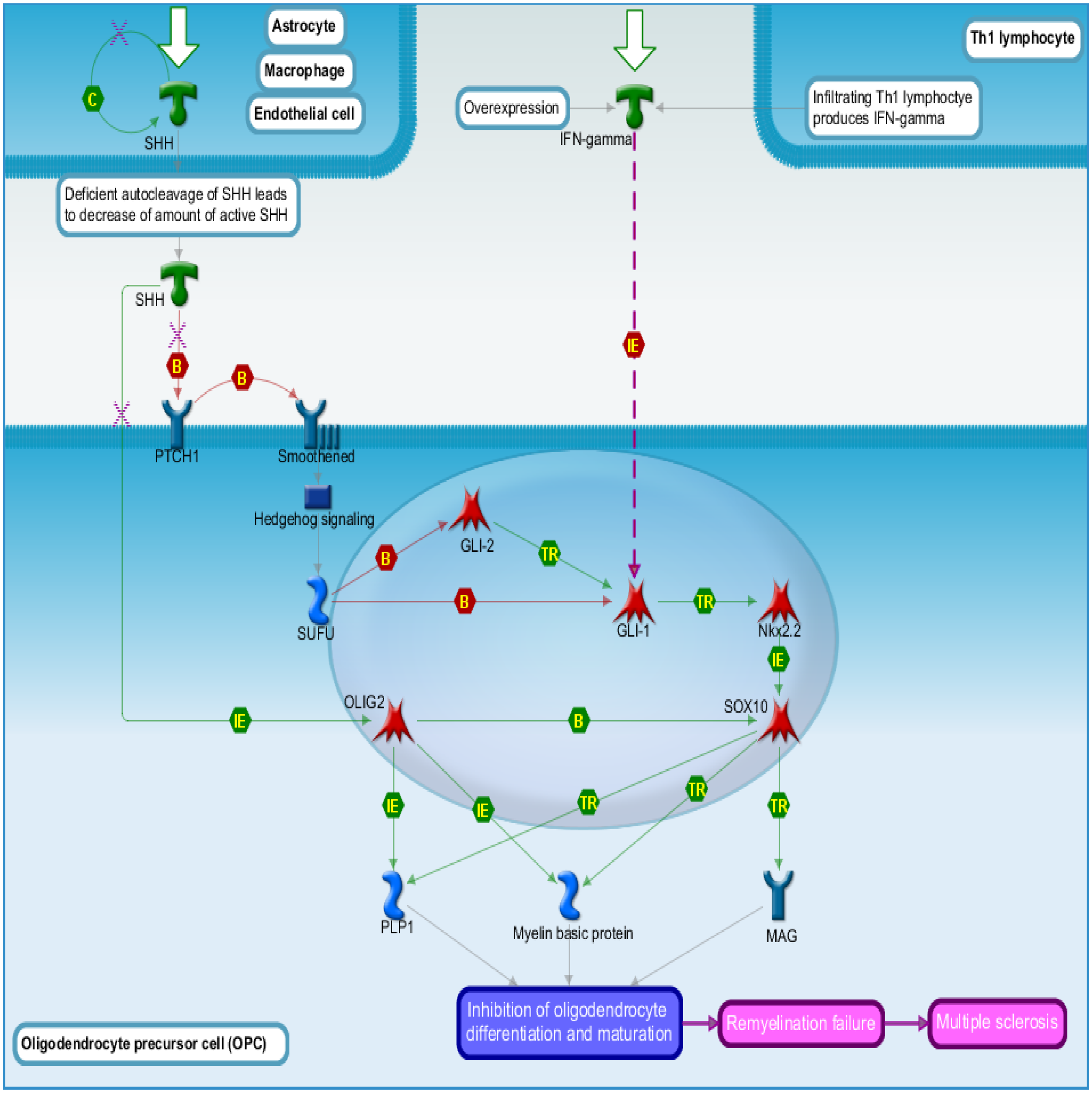

Figure 2: Sonic Hedgehog signaling in oligodendrocyte precursor cells differentiation in multiple sclerosis (DM 3305).

C.3 Disease map 4455

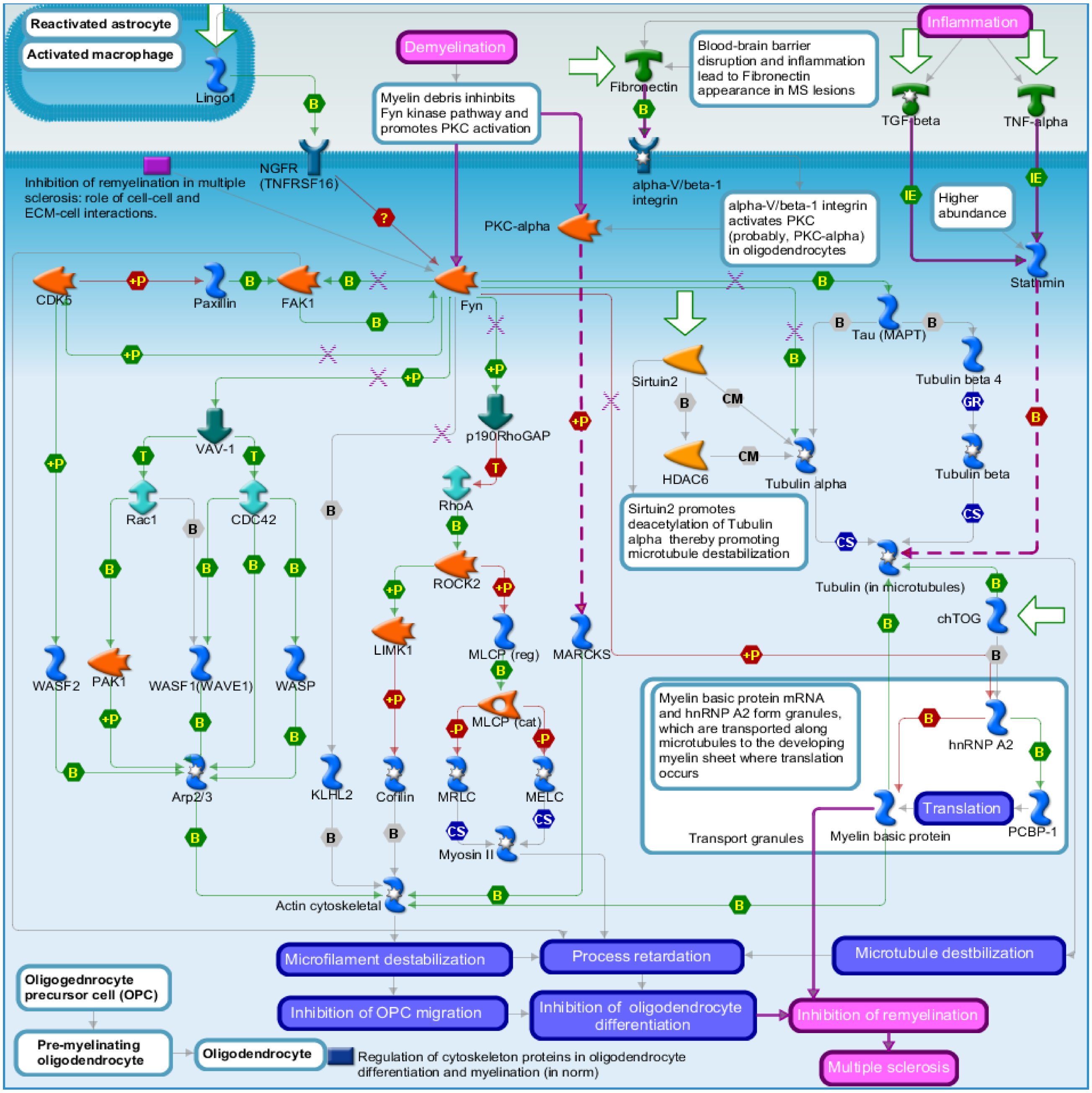

Figure 3: Inhibition of remyelination in multiple sclerosis: regulation of cytoskeleton proteins (DM 4455).

C.4 Disease map 5199

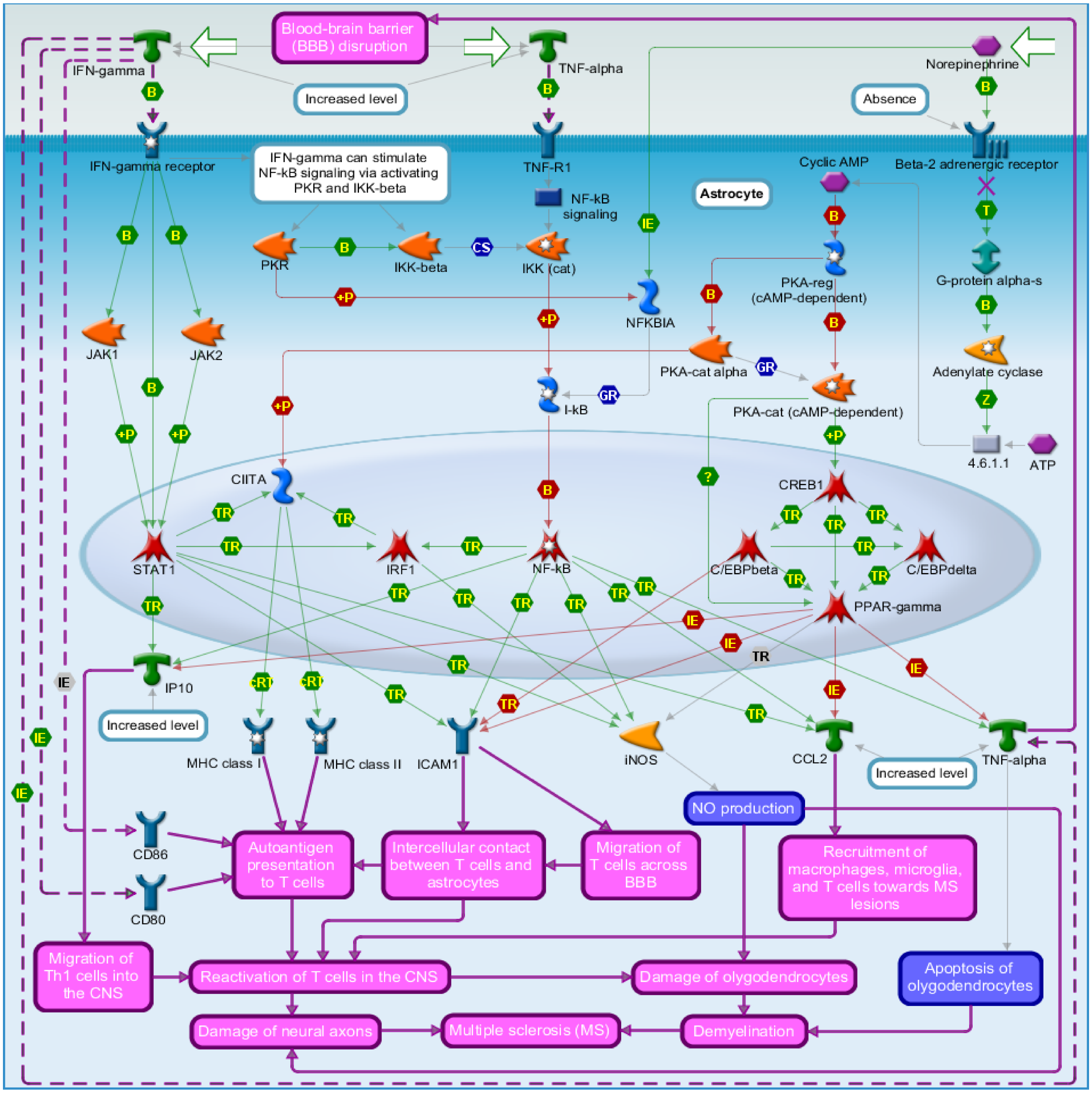

Figure 4: Cooperative action of IFN-gamma and TNF-alpha on astrocytes in multiple sclerosis (DM 5199).

D eQTL mapping

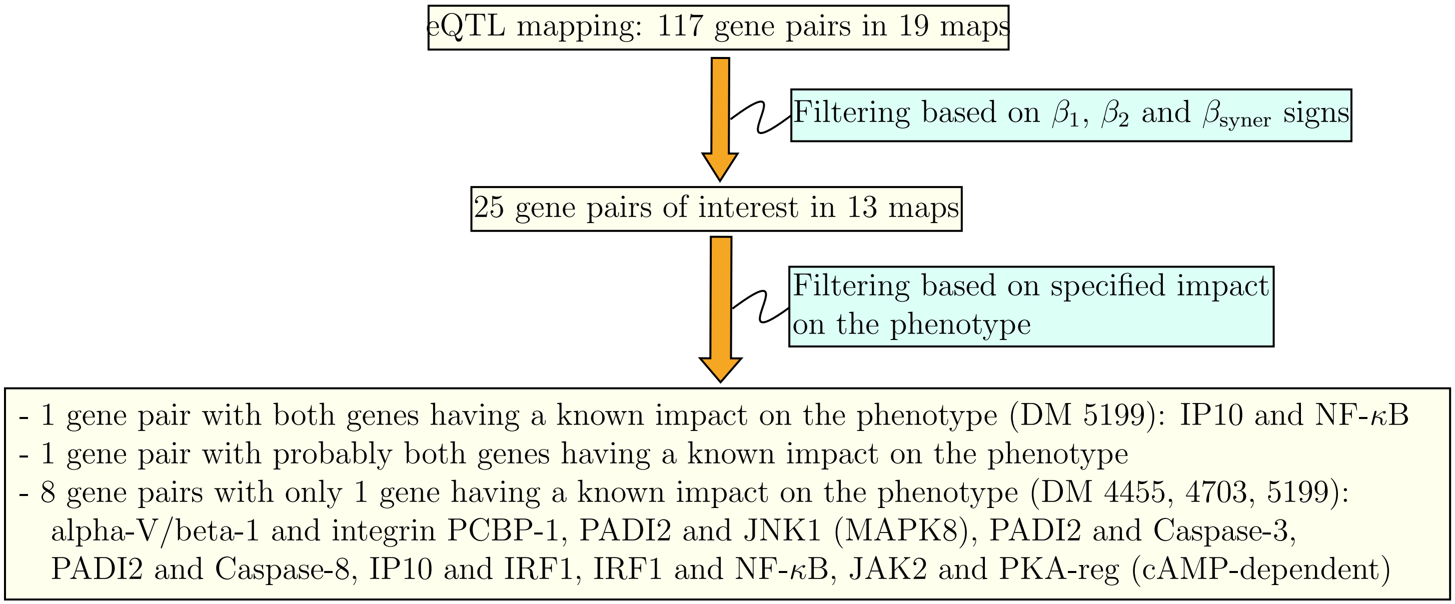

Figure 5: Filtering process for gene pairs identified by eQTL mapping.

Table 1: Compiled results of gene pairs identified by epistasis, and filtered according to the scheme in Supplementary Figure 5, with their specified or unknown impact on MS.

internal ID	Title	Interacting gene pair		β_x	β_y	β_{syner}	Specified impact on MS (activation or inhibition)
3302	Notch signaling in oligodendrocyte precursor cell differentiation in MS	RBP-J kappa (CBF1)	ADAM17	1.40	1.37	0.02	no
3306	Inhibition of oligodendrocyte precursor cells differentiation by Wnt signaling in MS	Beta-catenin	GSK3 beta	1.27	1.84	0.00	no
4455	Inhibition of remyelination in MS: regulation of cytoskeleton proteins	alpha-V/beta-1 integrin	PCBP-1	1.27	0.96	0.01	probably yes for alpha-V/beta-1 integrin
4703	Demyelination in MS	PADI2	JNK1(MAPK8)	-1.42	-1.58	-0.02	PADI2 enhances in disease
4703	Demyelination in MS	PADI2	Caspase-3	-1.56	-1.96	-0.01	PADI2 enhances in disease
4703	Demyelination in MS	PADI2	Caspase-8	-1.47	-1.21	-0.03	PADI2 enhances in disease
4703	Demyelination in MS	JNK1(MAPK8)	Caspase-8	-1.58	-1.21	-0.01	no
4791	Role of CNTF and LIF in regulation of oligodendrocyte development in MS	IMPA1	STAT3	1.41	1.10	0.02	no
4791	Role of CNTF and LIF in regulation of oligodendrocyte development in MS	PI3K reg class IA	STAT3	1.40	1.10	0.05	no
4843	Growth factors in regulation of oligodendrocyte precursor cells proliferation in MS	alpha-V/beta-3 integrin	SHP-2	1.34	1.97	0.07	no
4843	Growth factors in regulation of oligodendrocyte precursor cells proliferation in MS	SHP-2	c-Raf-1	1.63	1.63	0.09	no
4846	Growth factors in regulation of oligodendrocyte precursor cells survival in MS	ErbB2	Neuregulin 1	1.10	1.58	0.11	no
4846	Growth factors in regulation of oligodendrocyte precursor cells survival in MS	Neuregulin 1	Bcl-XL	-1.49	-1.17	-0.02	no
4901	Inhibition of remyelination in MS: role of cell-cell and ECM-cell interactions	Fyn	HYAL3	-1.99	-1.38	-0.07	no
5199	Cooperative action of IFN-γ and TNF-α on astrocytes in MS	IP10	IRF1	1.41	1.12	0.09	yes for IP10
5199	Cooperative action of IFN-γ and TNF-α on astrocytes in MS	IP10	NF-κB	1.39	0.98	0.09	yes for both genes
5199	Cooperative action of IFN-γ and TNF-α on astrocytes in MS	IRF1	NF-κB	1.16	0.88	0.07	yes for NF-κB
5199	Cooperative action of IFN-γ and TNF-α on astrocytes in MS	JAK2	PKA-reg (cAMP-dependent)	1.14	1.25	0.02	yes for JAK2
5288	Impaired inhibition of Th17 cell differentiation by IFN-beta in MS	IL-1RI	ROR-alpha	-1.16	-1.29	-0.09	yes (probable)
5398	Role of IFN-beta in activation of T cell apoptosis in MS	CTLA-4	TRADD	-1.61	-2.61	-0.04	no
5398	Role of IFN-beta in activation of T cell apoptosis in MS	Caspase-3	TRADD	-1.96	-2.21	-0.07	no
5518	Role of IFN-beta in inhibition of Th1 cell differentiation in MS	IFN-γ	PI3K reg class IA	1.29	1.40	0.07	no
5518	Role of IFN-beta in inhibition of Th1 cell differentiation in MS	GSK3 beta	IL-18R1	1.39	1.36	0.02	no
5518	Role of IFN-beta in inhibition of Th1 cell differentiation in MS	PI3K reg class IA	CD86	-0.96	-1.13	-0.18	no
5601	IL-2 as a growth factor for T cells in MS	GSK3 beta	Bcl-XL	-0.85	-1.17	-0.03	no
